# Multiple contact zones and karyotypic evolution in a neotropical frog species complex

**DOI:** 10.1038/s41598-024-51421-z

**Published:** 2024-01-11

**Authors:** Lucas H. B. Souza, Todd W. Pierson, Renata O. Tenório, Juan M. Ferro, Kaleb P. Gatto, Bruno C. Silva, Gilda V. de Andrade, Pablo Suárez, Célio F. B. Haddad, Luciana B. Lourenço

**Affiliations:** 1 Laboratório de Estudos Cromossômicos (LabEsC), Departamento de Biologia Estrutural e Funcional, Instituto de Biologia, Universidade Estadual de Campinas (UNICAMP), Campinas, SP 13083-863 Brazil; 2 Department of Ecology, Evolution, and Organismal Biology, Kennesaw State University, Kennesaw, GA USA; 3 Laboratorio de Genética Evolutiva “Dr. Claudio J. Bidau”, Instituto de Biología Subtropical (CONICET-UNaM), Facultad de Ciencias Exactas, Químicas y Naturales, Universidad Nacional de Misiones, Posadas, Misiones Argentina; 4 Departamento de Biologia, Centro de Ciências Biológicas e da Saúde, Universidade Federal do Maranhão (UFMA), Campus do Bacanga, São Luís, MA 65080-040 Brazil; 5 Instituto de Biología Subtropical (CONICET-UNaM), Puerto Iguazú, Argentina; 6 Departamento de Biodiversidade and Centro de Aquicultura (CAUNESP), Instituto de Biociências, Universidade Estadual Paulista, Rio Claro, SP Brazil

**Keywords:** Chromosomes, Population genetics, Speciation, Cytogenetics

## Abstract

Previous studies of DNA sequence and karyotypic data have revealed high genetic diversity in the *Physalaemus cuvieri – Physalaemus ephippifer* species complex—a group of small leptodactylid frogs in South America. To date, seven major genetic lineages have been recognized in this group, with species delimitation tests supporting four to seven of them as valid species. Among these, only *P. ephippifer* shows heteromorphic sex chromosomes, but the implications of cytogenetic divergence for the evolution of this group are unknown. We analyzed karyotypic, mitochondrial DNA, and 3RAD genomic data to characterize a putative contact zone between *P. ephippifer* and *P. cuvieri* Lineage 1, finding evidence for admixture and karyotypic evolution. We also describe preliminary evidence for admixture between two other members of this species complex—Lineage 1 and Lineage 3 of *P. cuvieri*. Our study sheds new light on evolutionary relationships in the *P. cuvieri – P. ephippifer* species complex, suggesting an important role of karyotypic divergence in its evolutionary history and underscoring the importance of hybridization as a mechanism of sex chromosome evolution in amphibians.

## Introduction

Speciation is a central topic in evolutionary biology, and hybridization between divergent lineages may impact speciation in several ways (for reviews, see Refs.^1–4^). Briefly, hybridization can break down species boundaries, counteracting speciation (e.g. Ref.^4^). Alternatively, if hybrids have reduced fitness, natural selection may favor interspecific divergence and reproductive isolation through reinforcement of species boundaries^5,6^. In some intermediate cases, backcrossing of viable hybrids with parental species may result in introgression (e.g. Ref.^7^). Finally, hybridization can generate a hybrid lineage that is reproductively isolated from its parental taxa, thus originating a new species through hybrid speciation (e.g. Ref.^8^).

As hybridization generates admixed genomes, it can blur species boundaries, thus representing a potential problem for species delimitation and taxonomy (for a review, see Ref.^9^). Although rival species concepts have been debated for decades^10–12^, a common element of most contemporary concepts is an emphasis on the independent evolutionary trajectory of each species^13^. In this sense, species can be recognized as separately evolving lineages, following the “general lineage concept of species” (Ref.^14^; but see Ref.^11^), and genetic analyses have been commonly incorporated in species delimitation studies of diverse organisms (e.g. Ref.^15–17^). However, because hybridization and introgression are more common than previously assumed (for reviews, see Refs.^1,9,18,19^), the identification of genetic lineages and the assessment of their reproductive isolation are often challenging, necessitating careful analysis of comprehensive multilocus datasets to discern the evolutionary processes at play. For instance, cytonuclear discordance (conflicting genetic patterns in nuclear vs mitochondrial markers) is common in hybridization and introgression scenarios (for a review, see Ref.^20^), but can also result from incomplete lineage sorting in the absence of gene flow^19,21^. Thus, specific analyses—such as the ABBA-BABA test, which identifies excess allele sharing between taxa due to hybridization^22^—have been widely used to assess the relative roles of gene flow versus incomplete lineage sorting (e.g. Ref.^23,24^).

Here, we investigated the *Physalaemus cuvieri – Physalaemus ephippifer* species complex, a group of South American leptodactylid frogs that exhibits high genetic diversity and unresolved taxonomic issues^25^. Based on DNA sequences (mitochondrial DNA and 3RAD markers) and karyotypes, Nascimento et al.^25^ recognized seven major genetic lineages in this group, and species delimitation tests based on DNA sequences supported four to seven of these lineages as potential species. One of the uncertainties regarding species delimitation in this group involved a clade composed of *P. ephippifer* and Lineage 1 of *P. cuvieri *sensu lato (hereafter L1). The delimitation test based on 3RAD markers (BPP test) recovered *P. ephippifer* and L1 as distinct species, while the bPTP test based on mitochondrial DNA markers provided only partial support for the recognition of both lineages as different species^25^. However, this previous study was limited by relatively sparse geographic sampling and the absence of morphological or acoustic data, thus prompting further work to robustly characterize species boundaries.

Interestingly, heteromorphic sex chromosomes Z and W are present in *P. ephippifer*^26^ but not in L1 or in any other lineage in the species complex^25,27^. The W chromosome of *P. ephippifer* differs from its Z chromosome mainly through the presence of an additional nucleolar organizer region (NOR) and a distal heterochromatic band in the short arm^26^, but also by the presence of a larger site enriched in the satellite DNA PcP190 in the long arm^28^. Chromosome 9 of the L1 karyotype has been inferred to be homeologous to the sex chromosomes of *P. ephippifer* based on the presence of NORs that coincide with heterochromatic C-band enriched in the repetitive DNA PepBS^29^. This inference was further supported by the detection of a pericentromeric region of the short arm of chromosome 9 using a probe constructed from the microdissection of the pericentromeric region of the long arm of the *P. ephippifer* Z chromosome (the Zqper probe)^29^. In addition to the intrachromosomal location of the region detected by the Zqper probe, chromosome 9 of L1 differs from both the Z and W chromosomes of *P. ephippifer* by having a smaller short arm^29^. Although chromosome 9 of L1 is polymorphic with respect to NOR size and number, no sex-related heteromorphism has been reported in this lineage^27,29^.

Sex chromosomes are known to play important roles in speciation, as the presence of distinct sex chromosome systems may lead to reproductive barriers^30–33^. However, the extent of hybridization between *P. ephippifer* and L1 and the potential role of cytogenetic differences in their divergence remain unexplored. To address this, we analyzed specimens from sites located near the distribution boundaries of *P. ephippifer* and L1 and near their putative contact zone using karyotypic, mitochondrial, and 3RAD genomic data.

## Results

**Figure 1:**
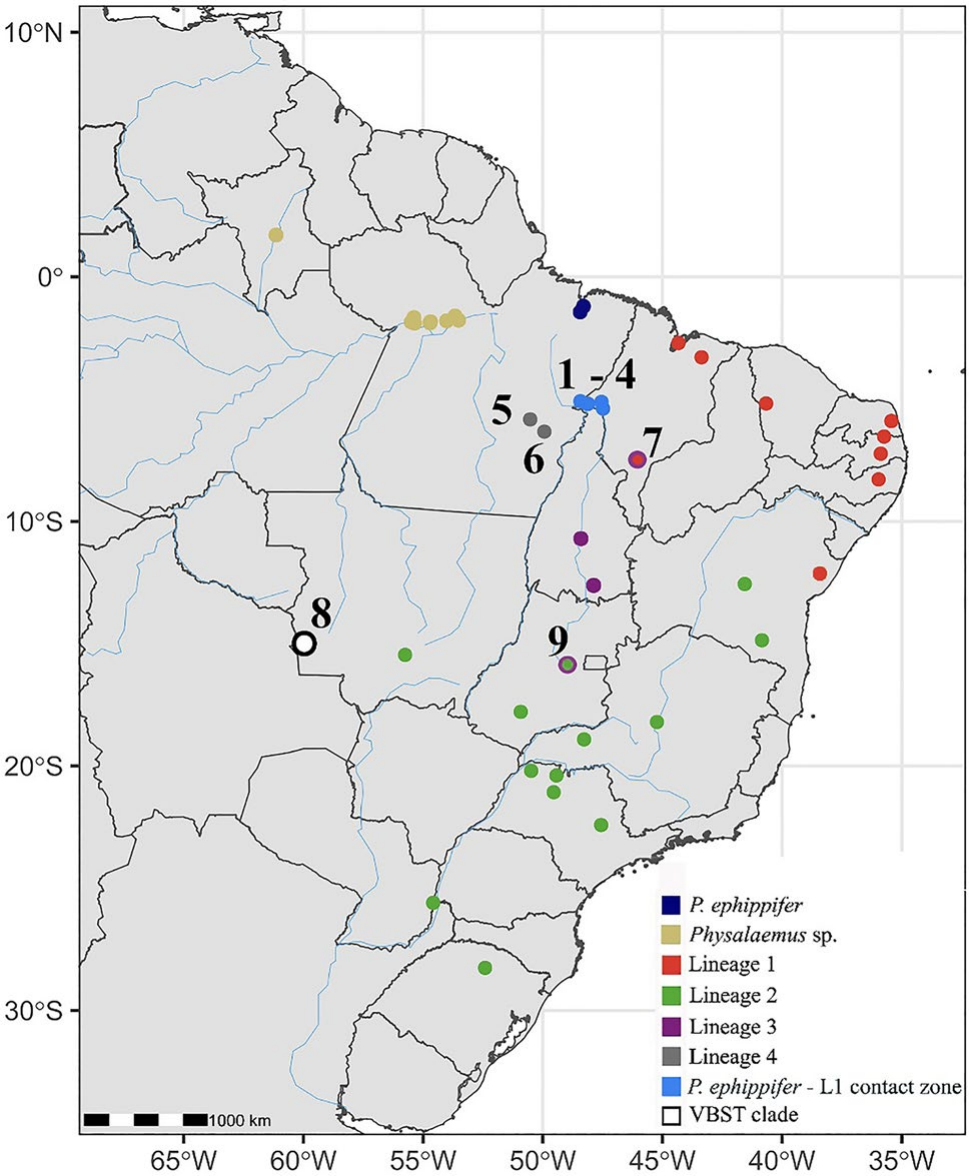
Geographic distribution of the *Physalaemus cuvieri – P. ephippifer* species complex. The numbers 1 – 6 indicate sampled sites near the range boundaries of *Physalaemus ephippifer* and the L1 lineage of *P. cuvieri* and their putative contact zone (Lourenço et al.^57^; Nascimento et al.^25^). 1. São Pedro da Água Branca (SPAB), State of Maranhão. 2. Vila Nova dos Martírios (VNM), State of Maranhão. 3. São Francisco do Brejão—Trecho Seco (TS), State of Maranhão. 4. Imperatriz (Imp), State of Maranhão. 5. Marabá (Mar), State of Pará (L4 lineage – see description below). 6. Parauapebas (Par), State of Pará (L4 lineage – see description below). 7. Balsas (Bal), State of Maranhão. 8. Vila Bela da Santíssima Trindade (VBST), State of Mato Grosso (VBST clade – see description below). 9. Pirenópolis (Pir), State of Goiás (contact between L2 and L3). Map generated using the sf and geobr packages in R v4.1.0^84^, and edited in Adobe Photoshop CC v. 2017.1.1.

Our analyses included 37 specimens from the geographic area of primary interest in this study, which comprises the distribution boundaries of *P. ephippifer* and L1 and their potential contact zone. This sampled area includes six localities, numbered 1–6 in Fig. 1, respectively: São Pedro da Água Branca (SPAB, admixed *P. ephippifer* x L1), Vila Nova dos Martírios (VNM, admixed *P. ephippifer* x L1), São Francisco do Brejão—Trecho Seco (TS, admixed *P. ephippifer* x L1), Imperatriz (Imp, admixed *P. ephippifer* x L1), Marabá (Mar, assigned to L4), and Parauapebas (Par, assigned to L4). Additionally, we included individuals from Vila Bela da Santíssima Trindade (VBST, site 8 in Fig. 1), a locality situated in west-central Brazil, Balsas (Bal, site 7 in Fig. 1), and Pirenópolis (Pir, site 9 in Fig. 1). We also expanded the specimen sampling of *P. ephippifer*, L1, L2, and L3 to 13, 27, 26, and 9 individuals, respectively. For detailed information on samples used in each analysis, refer to Supplementary Table S1.

### Mitochondrial DNA analyses

**Figure 2:**
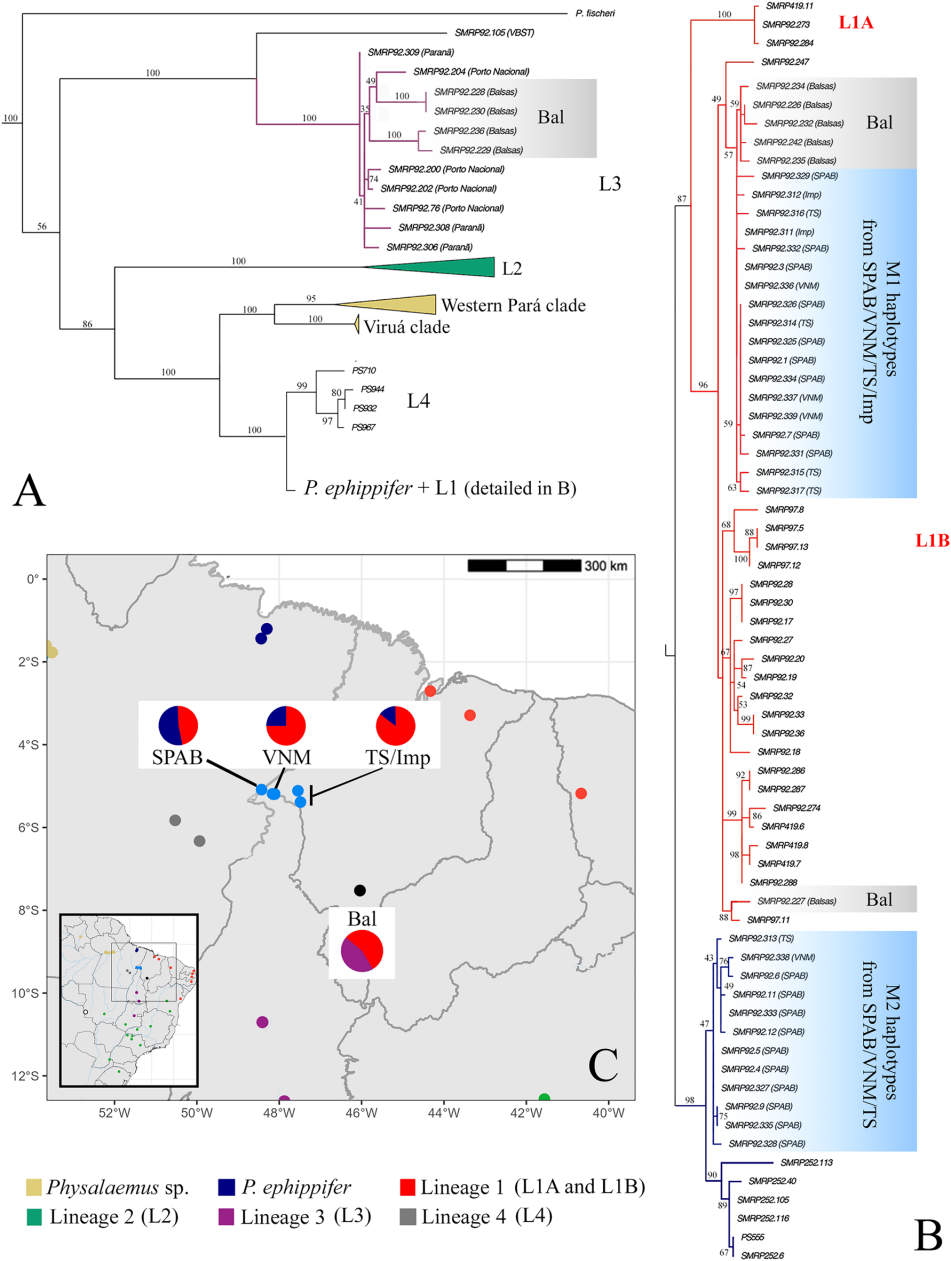
Phylogenetic relationships of the *Physalaemus cuvieri – P. ephippifer* species complex inferred by RAxML analysis of mitochondrial DNA sequences. (**A**) Overview of the internal relationships in the *P. cuvieri – P. ephippifer* species complex. *Physalaemus fischeri* is the only representative of the outgroup shown in this figure. Note that some samples from Bal (gray rectangle) are clustered within L3. The complete cladogram is available in Supplementary Fig. S1. A finer resolution of the branch labeled *P. ephippifer* + L1 is shown in (**B**). (**B**) Phylogenetic relationships based on mtDNA sequences from samples collected from SPAB, VNM, TS, Imp (blue rectangles in the cladogram), and Bal (gray rectangles in the cladogram), whose geographic locations are depicted on the map shown in (**C**). Numbers on cladogram branches represent bootstrap values. In (**C**), the relative frequencies of haplotypes that cluster with *P. ephippifer* (blue), L1 (red), or L3 (purple) haplotypes are shown for SPAB, VNM, TS/Imp, and Bal (sites 1–4 and 7 in Fig. 1). Map generated using the sf and geobr packages in R v4.1.0^84^, and edited in Adobe Photoshop CC v. 2017.1.1.

In our phylogenetic analysis of the mtDNA dataset, we recovered all major genetic lineages previously recognized in the *P. cuvieri – P. ephippifer* species complex, including *P. ephippifer*, *Physalaemus* sp. (Western Pará and Viruá clades), L1 (composed of L1A and L1B subclades), L2, and L3 (Fig. 2A; Supplementary Fig. S1). At SPAB, VNM, and TS, we found two distinct haplogroups in syntopy (M1 and M2). The M1 haplogroup included sequences of nine specimens from SPAB, three from VNM, four from TS, and all specimens from Imp and Bal. This M1 haplogroup was nested within the L1B clade. In contrast, the M2 haplogroup included sequences of 10 specimens from SPAB, 1 from VNM, and 1 from TS, and was sister to the *P. ephippifer* clade (Fig. 2B). We estimated a genetic distance of 1.12% between M1 and M2 haplogroups using the MVZ59–16Sbr fragment, and of 1.39% using only the 16Sar–16Sbr fragment (Table 1).

**Table 1:** Uncorrected p-distances (%) based on the mitochondrial H1 fragment (2300 bp; upper-right triangular) and 16Sar-16Sbr segment (546 bp; lower-left triangular).

	*P. ephippifer*	M2 haplogroup	L1B	M1 haplogroup	L1A	Balsas (L1)	Balsas (L3)	L3	VBST	L4	*Physalaemus* sp.	L2
*P. ephippifer*	**0.25/0.11**	0.46	1.40	1.27	1.50	1.36	5.46	5.04	5.65	1.27	2.16	4.92
M2 haplogroup	0.65	**0.24/0.14**	1.31	1.12	1.33	1.23	5.15	4.80	5.35	1.14	1.99	4.69
L1B	1.49	1.54	**0.53/0.53**	0.61	1.31	0.65	5.10	4.66	5.25	1.47	2.42	4.72
M1 haplogroup	1.45	1.39	0.54	**0.12/0.12**	1.16	0.27	5.12	4.72	5.13	1.29	2.29	4.58
L1A	1.61	1.61	1.41	1.36	**0/0.06**	1.24	5.54	5.06	5.31	1.53	2.56	4.90
Balsas (L1)	1.55	1.47	0.60	0.25	1.43	**0.35/0.33**	5.24	4.83	5.19	1.37	2.41	4.69
Balsas (L3)	4.75	4.70	4.61	4.47	5.39	4.59	**1.05/0.65**	0.77	3.21	5.34	5.40	6.02
L3	4.24	4.23	4.27	4.23	4.88	4.34	1.09	**0.50/0.39**	2.96	4.97	4.98	5.45
VBST	5.15	5.03	5.25	5.07	5.40	5.17	3.19	2.97	**–/–**	5.37	5.46	5.89
L4	1.43	1.42	1.42	1.38	1.49	1.43	5.18	4.78	5.40	**0.19/0.37**	2.15	4.73
*Physalaemus* sp.	2.29	2.32	2.66	2.73	2.85	2.80	4.22	3.79	4.94	2.63	**0.79/1.43**	4.74
L2	6.19	6.52	6.54	6.55	7.30	6.64	6.28	5.62	5.96	6.33	5.97	**1.4/1.23**

In bold (diagonal) the uncorrected p-distances (%) estimated within each sequence group using the H1 (right) and 16Sar-16Sbr (left) fragments. M1 haplogroup: samples from SPAB-VNM-TS-Imp that grouped together with L1 haplotypes in the phylogenetic analysis (see Fig. 2). M2 haplogroup: samples from SPAB-VNM-TS that grouped together with *P. ephippifer* in the phylogenetic analysis (see Fig. 2). Balsas (L1): samples from Balsas that clustered with L1 in the phylogenetic analysis (see Fig. 2). Balsas (L3): samples from Balsas that clustered within L3 in the phylogenetic analysis (see Fig. 2). L1A, L1B, L2, L3, and L4: lineages of “*P. cuvieri*”. *Physalaemus* sp.: samples included in the Western Pará and Viruá clades of Nascimento et al.^25^.

At locality Bal, we found haplotypes typical of L3 in addition to haplotypes of L1 (Fig. 2, Supplementary Figs. S1, S2). Six specimens from this locality clustered within the L1 clade, while four other specimens were nested in the L3 clade, together with specimens from Porto Nacional (State of Tocantins) and Paranã (State of Tocantins) (Fig. 2). It is worth noting that in this analysis, the L3 clade was sister to the specimen from Vila Bela da Santíssima Trindade (VBST) (State of Mato Grosso). The mitochondrial haplotype found in VBST, however, was notably divergent from the remaining sequences in L3, as shown by the phylogenetic analysis (Fig. 2A), the haplotype network (Supplementary Fig. S2), and the genetic distance analyses (Table 1).

Finally, mtDNA analyses revealed a previously unrecognized lineage composed of specimens from Mar and Par, which we refer to as Lineage 4 (L4) (Fig. 2). The L4 clade was strongly supported (bootstrap value = 99%), as was the clade composed of L4, L1 (including the M1 haplogroup), and *P. ephippifer* (including the M2 haplogroup) (bootstrap value = 100%), (Fig. 2). L4 was inferred to be sister to the clade composed of L1 and *P. ephippifer*, although this latter clade was not supported by the bootstrap analysis (Fig. 2). We estimated a genetic distance of 1.48% between L4 and L1 (including L1A and L1B) using the MVZ59–16Sbr fragment and of 1.43% using only the 16Sar–16Sbr fragment. Between L4 and *P. ephippifer*, we found a divergence of 1.27% in the MVZ59–16Sbr fragment and of 1.43% in the 16Sar–16Sbr fragment (Table 1).

### 3RAD data assembly and analyses

Our full 3RAD assembly included > 230,000 loci. Of these, > 200,000 loci were variable and contained a total of > 2.7 million SNPs. This final assembly included between 13,468 and 45,449 loci per individual (Supplementary Table S2).

**Figure 3:**
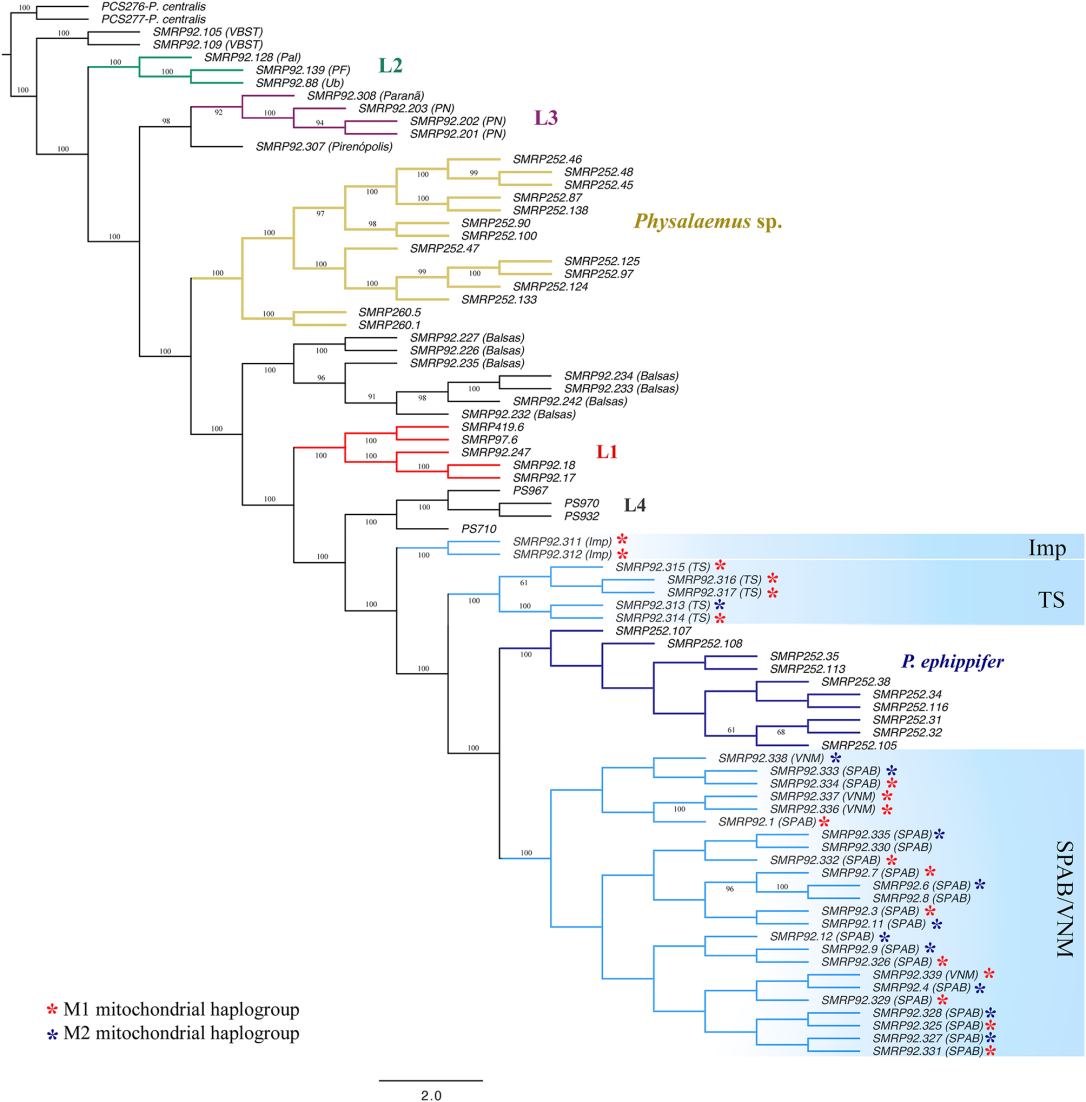
RAxML cladogram inferred from the 3RAD dataset including one random SNP per locus (83 individuals, 205,030 SNPs). Asterisks indicate the mitochondrial haplogroups to which the sampled specimens from SPAB, VNM, TS, and Imp belong.

The maximum-likelihood cladograms inferred from the full 3RAD dataset and the dataset that included one random SNP per locus were quite congruent, with the few differences regarding relationships among individuals within specific lineages. In both 3RAD cladograms*,* we recovered samples from VBST, then L2, and then L3 as sequentially sister to the remaining ingroup samples (Fig. 3). In contrast to the analysis based on mtDNA, analyses of the 3RAD dataset recovered all the specimens from Bal (n = 7) as a clade, which was sister to a clade consisting of L1, L4, *P. ephippifer*, and all samples from the putative contact zone between L1 and *P. ephippifer*. Focusing on this contact zone, our analysis inferred a clade composed of samples from TS (n = 5), a clade composed of samples from Imp (n = 2), and a clade composed of samples from SPAB (n = 19) and VNM (n = 4)—with this last clade recovered as sister to *P. ephippifer*. Similar to the mtDNA results, the phylogenetic analysis of the 3RAD dataset recovered a clade corresponding to L4 (i.e. the samples from Mar and Par), a clade corresponding to L3, and a clade corresponding to *Physalaemus* sp. (Western Pará and Viruá clades). Another discordance between mtDNA and 3RAD data involved the placement of SMRP 92.307 from locality Pir, which did not group with L2 but was recovered as sister of L3 instead (Fig. 3; Supplementary Fig. S3).

The NeighborNet network inferred from 3RAD data (Supplementary Fig. S4) showed results that were largely concordant with those from the RAxML phylogenetic inference. For example, all samples from Bal clustered with each other but separate from L1. Additionally, we recovered the same major groups within the contact zone between *P. ephippifer* and L1, with samples from SPAB and VNM being closer to *P. ephippifer* than the samples from TS and Imp. Furthermore, we recovered SMRP 92.307 from Pirenópolis as separate from L3, which was concordant with our RAxML phylogenetic inference (Fig. 3; Supplementary Fig. S3). However, in the phylogenetic network, we recovered SMRP 92.307 with SMRP 92.308 (from Paranã), which was clustered in L3 in the RAxML analysis (Fig. 3; Supplementary Fig. S3).

**Figure 4:**
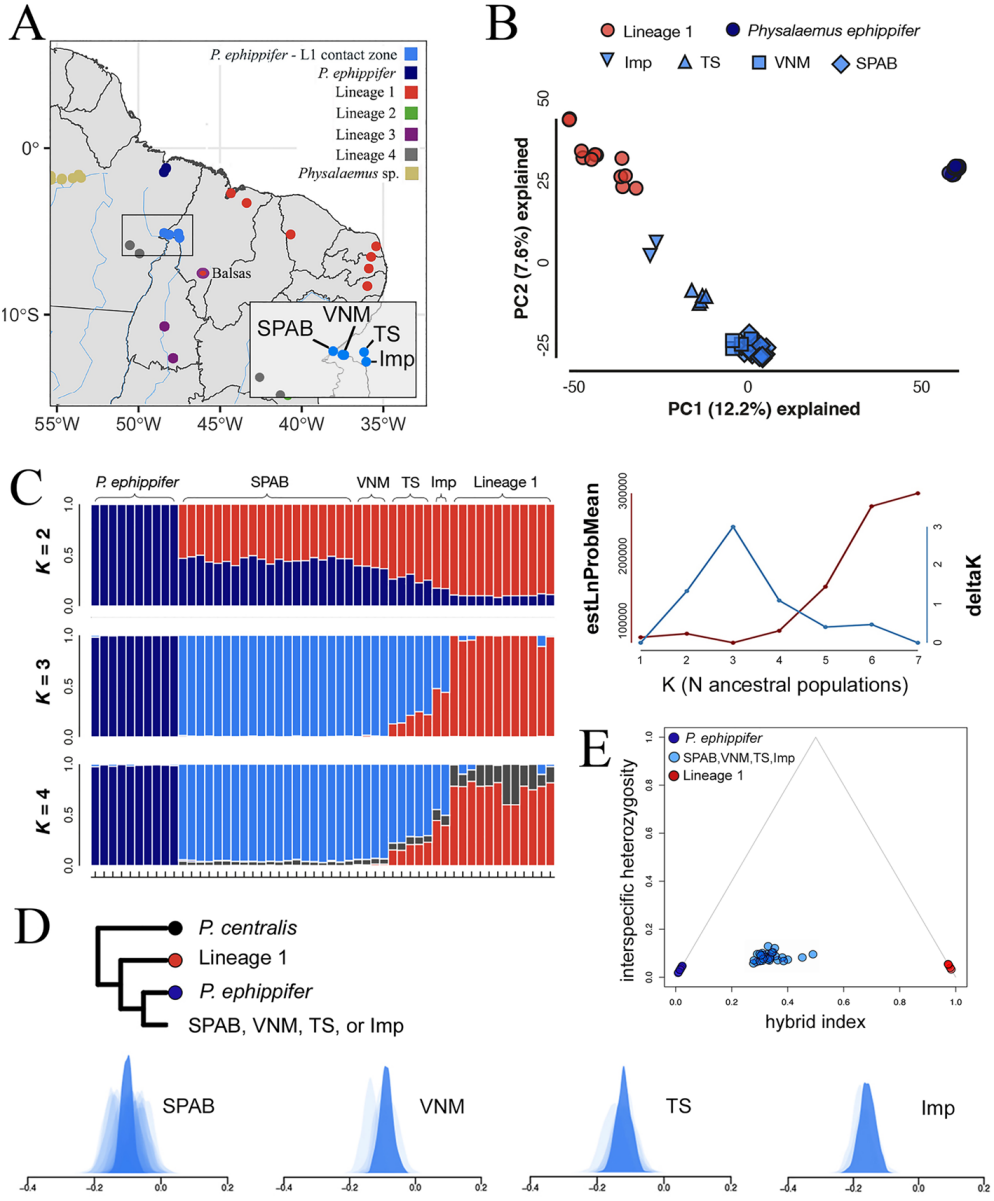
Comparative analyses of *P. ephippifer*, L1, and the specimens from SPAB, VNM, TS, and Imp based on the 3RAD dataset. (**A**) Map showing the geographic distribution of lineages in the *Physalaemus cuvieri – P. ephippifer* species complex. In the inset, a larger scale map of the region that includes SPAB, VNM, TS, and Imp. Map generated using the sf and geobr packages in R v4.1.0^84^, and edited in Adobe Photoshop CC v. 2017.1.1. (**B**) Principal component analysis (PCA) plot based on 53 individuals and 45,800 SNPs. (**C**) Results of Structure analyses showing ancestry probabilities of individuals in the *P. ephippifer* X L1 hybrid zone, based on 53 individuals and 3,465 SNPs. (**D**) ABBA-BABA tests for introgression between *P. ephippifer* and L1 in SPAB, VNM, TS, and Imp. Light shaded distributions show bootstrap replicates from individual samples, and dark shaded distributions show bootstrap replicates from pooled samples at each site. Dendrogram illustrates the hypothesized phylogenetic relationships used to establish each set of analyses. (**E**) Triangle plot showing the hybrid index and interspecific heterozygosity calculated for 10 individuals of *P. ephippifer*, 5 of L1, and 31 of the contact zone (i.e. 20 individuals from SPAB, 4 from VNM, 5 from TS, and 2 from Imp), using 406 SNPs.

**Figure 5:**
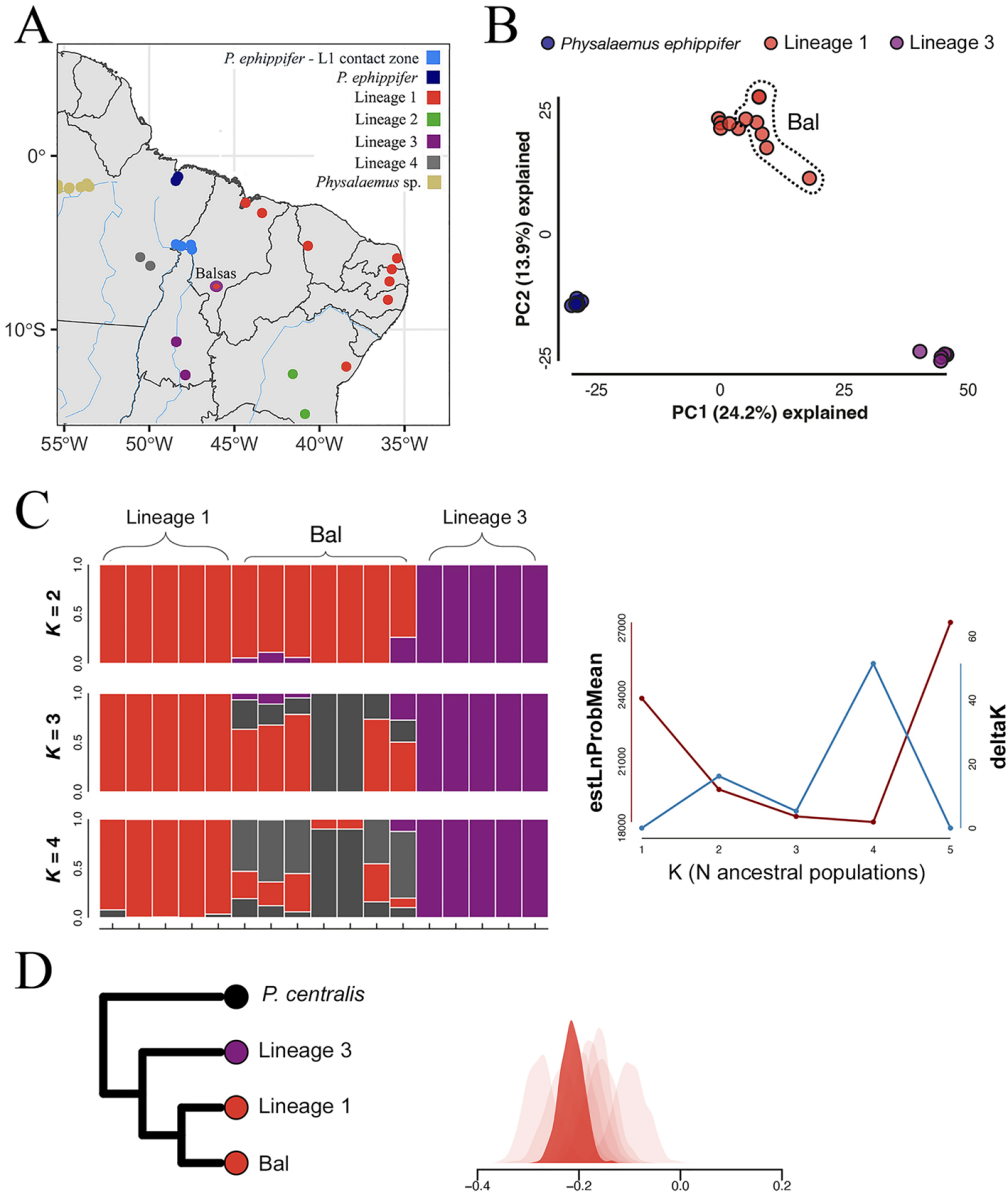
Comparative analyses of L1, L3, and the specimens from Bal based on the 3RAD dataset. (**A**) Map showing the geographic distribution of lineages in the *Physalaemus cuvieri – P. ephippifer* species complex, showing the location of Bal. Map generated using the sf and geobr packages in R v4.1.0^84^, and edited in Adobe Photoshop CC v. 2017.1.1. (**B**) Principal component analysis (PCA) plot based on 27 individuals and 14,782 SNPs. (**C**) Results of Structure analyses showing ancestry probabilities of individuals in the L1 X L3 hybrid zone, based on 17 individuals and 1,772 SNPs. (**D**) ABBA-BABA tests for introgression between L1 and L3 in Bal. Light shaded distributions show bootstrap replicates from individual samples, and dark shaded distributions show bootstrap replicates from pooled samples.

In the first PCA (i.e. the analysis that included all samples), PC1 separated samples from *Physalaemus* sp. (Western Pará and Viruá clades) from all other samples and explained 24.4% of all variation. The second PC axis (explaining 9.4% of all variation) separated the remaining samples into two major groups: one consisting of the outgroup *P. centralis*, VBST, L2, and L3, and one consisting of L4, *P. ephippifer*, L1, and the contact zone between *P. ephippifer* and L1 (Supplementary Fig. S5). In the second PCA, which focused on this contact zone, the first PC axis separated L1 from *P. ephippifer* and explained 12.2% of all variation (Fig. 4). All samples from the contact zone fell at intermediate positions on this first PC axis, with those from SPAB and VNM being closest to *P. ephippifer*, those from Imp being closest to L1, and those from TS being intermediate in position. The second PC axis explained 7.6% of variation and mostly separated samples from the contact zone from those in both “parental” lineages (i.e. *P. ephippifer* and L1). Finally, in the third PCA, which focused on evaluating evidence for gene flow between L3 and samples from Bal (in L1), the first PC axis (24.2% of variation) separated *P. ephippifer* from L3, with all samples from L1 occupying intermediate positions on this axis (Fig. 5). Notably, samples from Bal showed positions on this PC axis closer to L3 than any other samples in L1. The second PC axis (13.9% of variation) separated L1 from the other two groups.

In the first set of Structure analyses (Fig. 4), we found the strongest support for K = 3 and plotted posterior probabilities of population assignment (hereafter “assignment probabilities”) for K = 2–4. At K = 2, samples from *P. ephippifer* had uniformly high assignment probabilities to one cluster and samples from L1 had uniformly high assignment probabilities to the second cluster; samples from the contact zone had intermediate assignment probabilities to each cluster, with those from SPAB and VNM having relatively higher assignment probabilities to the cluster corresponding to *P. ephippifer*, those from Imp having relatively higher assignment probabilities to the cluster corresponding to L1, and those from TS falling in-between. At K = 3, samples from SPAB and VNM had high assignment probabilities to a third cluster, with samples from TS and Imp having assignment probabilities split between this third cluster and the cluster corresponding to L1. The analyses at K = 4 appear similar to those at K = 3 and failed to reveal any additional, meaningful population structure (Fig. 4). In the second set of Structure analyses (Fig. 5), we found some support for K = 2 and K = 4, and we plotted assignment probabilities fork K = 2–4. At K = 2, most samples from L1 had uniformly high assignment probabilities to one cluster and samples from L3 had uniformly high assignment probabilities to the second. Some samples from Bal had low, but non-zero, assignment probabilities to L3. Analyses at K = 3 and K = 4 revealed additional population structure among samples from Bal, with samples SMRP 92.233 and SMRP 92.234 appearing to be the most distinct (Fig. 5).

The ABBA-BABA tests focused on the contact zone between L1 and *P. ephippifer* revealed statistically significant and negative D-statistics (i.e. indicating more L1 alleles in these samples than in *P. ephippifer*) for all analyzed sampling sites. These D-statistics varied from − 0.157 (p < 0.01) for Imp to − 0.088 for VNM (p < 0.01) (Fig. 4D). The ABBA-BABA test focused on samples from Bal also revealed a significant negative D-statistic of − 0.214 (p < 0.01) (i.e. indicating more L3 alleles in Bal than in other populations from L1) (Fig. 5).

### Cline analyses of the contact zone between *P*. *ephippifer* and L1

**Figure 6:**
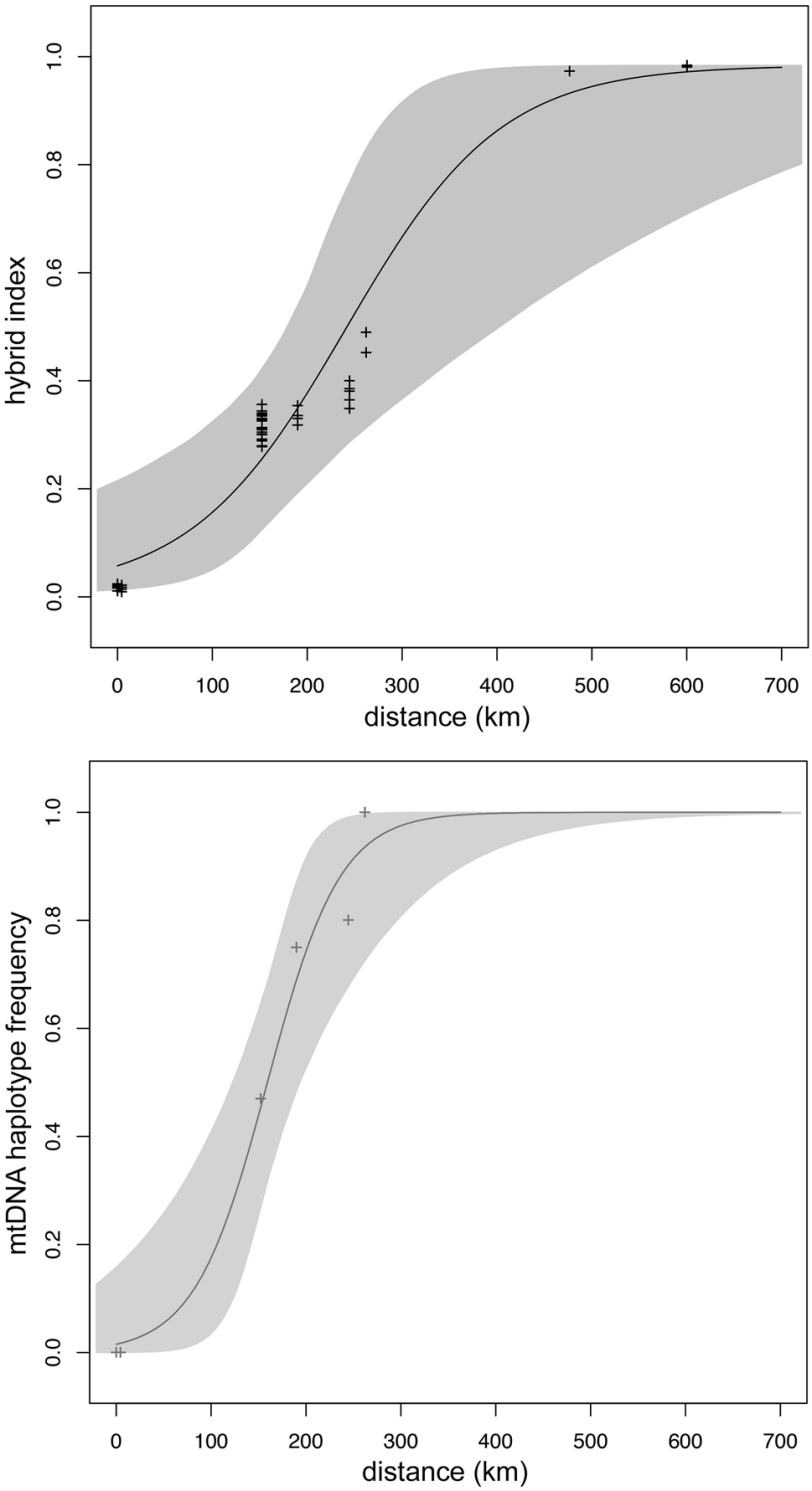
Cline analyses of hybrid indexes estimated from 3RAD dataset (top) and mitochondrial DNA haplotype frequencies (bottom) across the contact zone between *Physalaemus ephippifer* and L1 along a west–east transect. Shaded areas represent the 95% confidence interval of fitted clines.

In the cline analyses of the contact zone between *P. ephippifer* (hybrid index = 0) and L1 (hybrid index = 1) based on 3RAD data, we estimated mean hybrid indexes of 0.28 ± a standard deviation of 0.02 for SPAB, 0.29 ± 0.01 for VNM, 0.33 ± 0.2 for TS, and 0.42 ± 0.02 for Imp. All estimates of interspecific heterozygosity were low (range = 0.02–0.13) (Fig. 4E). In the geographic cline fit to the hybrid index, we estimated a cline center of 317 km (95% credible interval [CI]   188–596 km) and a cline width of 713 km (CI   219–1,800 km). In the geographic cline fit to the mtDNA haplotype frequencies, we estimated a cline center of 163 km (CI   102–253 km) and a cline width of 319 km (CI   100–1219 km) (Fig. 6).

### Cytogenetic analyses of specimens from SPAB and VNM

**Figure 7:**
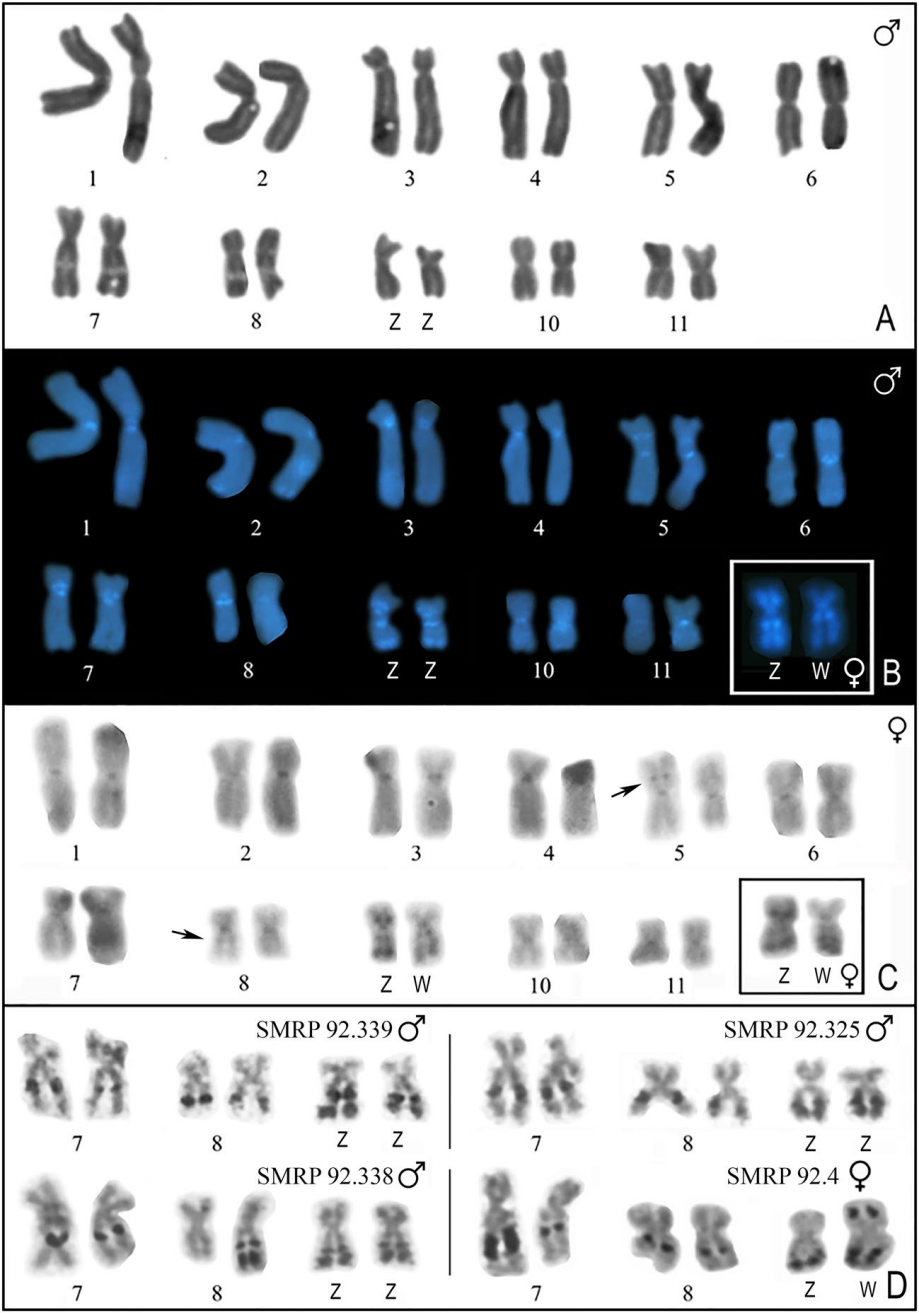
Karyotype of specimens from the contact zone between *Physalaemus ephippifer* and L1. (**A**,**B**) Giemsa-stained (**A**) and C-banded/DAPI-stained male karyotype (**B**). The inset in B shows a sex chromosome pair of a female specimen. (**C**) C-banded female karyotype. The inset in C shows a ZW pair from another metaphase, in which the C-bands of the W chromosome are easily seen. (**D**) Silver-stained NOR-bearing chromosomes of four individuals. Note the interindividual variation in NOR number/size.

All of the analyzed individuals from SPAB and VNM had a 2n = 22 karyotype, composed of five pairs of metacentric chromosomes and six pairs of submetacentric chromosomes (Fig. 7A–C). Chromosomes 7, 8, and 9 carried nucleolus organizer regions (NORs), as revealed by silver impregnation using the Ag-NOR method in metaphases of 13 individuals (Fig. 7D). In all of the analyzed specimens, chromosome pair 7 had interstitial NOR in the long arm. In four males and one female, pair 7 was heteromorphic with respect to the NOR size (Fig. 7).

Regarding chromosome pair 8, the presence of one interstitial NOR in the long arm of both homologues was the most frequent condition. However, in three of the 13 analyzed specimens (i.e. SMRP 92.1, SMRP 92.327, and SMRP 92.338), a single NOR-bearing chromosome 8 was present. In this latter specimen (SMRP 92.338), the NOR-bearing chromosome 8 had an additional distal NOR, also in the long arm (Fig. 7D). The variation regarding chromosome 8 was not sex-related.

In contrast, chromosome pair 9 was heteromorphic regarding the number of NORs exclusively in females, suggesting the presence of a ZZ/ZW chromosome system. In the ten males for which a NOR-pattern could be conclusively identified, both chromosomes 9 had only two NORs in the long arm (one interstitial and one terminal), while in the three females, one homologue of pair 9 had an additional distal NOR in the short arm (Fig. 7D).

The C-banding revealed heterochromatic bands in all centromeric regions and an interstitial band in the short arm of chromosome 5. A faint C-band adjacent to the NOR in chromosome 8 could be seen in some metaphases (Fig. 7C). Also, the NORs located in the long arm of chromosomes 9 (tentatively identified as Z and W chromosomes and hereafter referred to as such) coincided with heterochromatic bands (Fig. 7B,C). In addition, the Z chromosome had pericentromeric C-band in the short and long arms, whereas the W chromosome had a pericentromeric C band in the long arm (Fig. 7C). In the C-banded metaphases stained with DAPI, pericentromeric bands were visible in the short arm of chromosome 7 and in the long arm of chromosome 8 (Fig. 7B).

**Figure 8:**
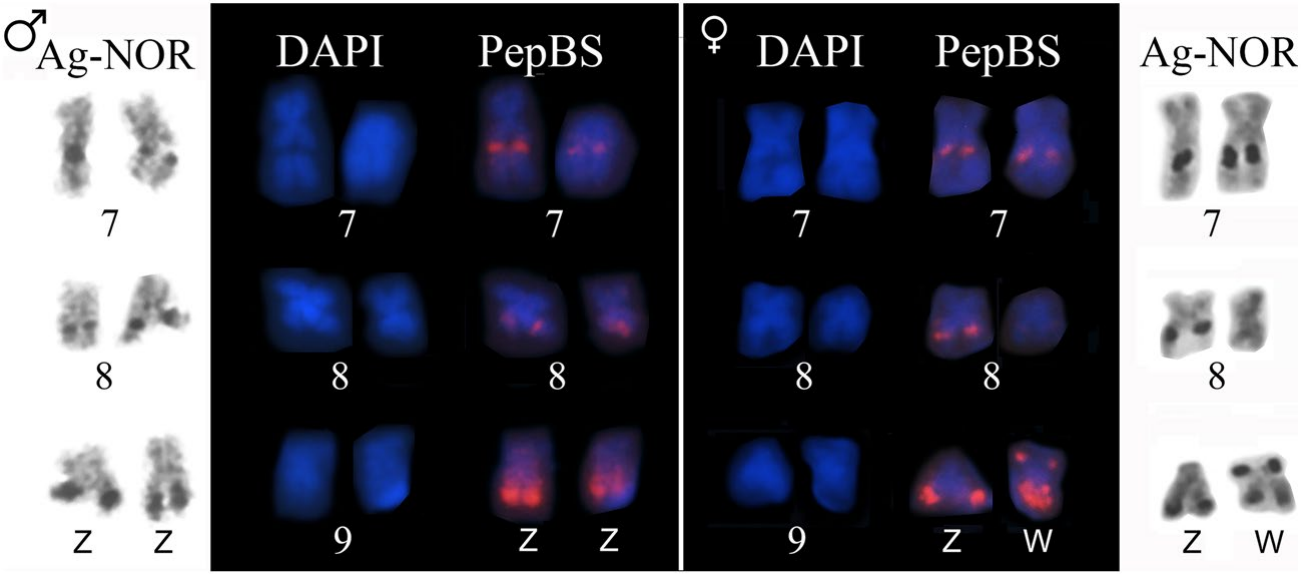
Characterization of the NOR-bearing chromosomes found in the contact zone between *Physalaemus ephippifer* and L1. NOR-bearing chromosomes of a male (left panel) and a female (right panel) specimen from SPAB subjected to the Ag-NOR method, DAPI-stained, and hybridized to a PepBS probe. Note the strong and diffuse probe signals in the long arm of the Z and W sex chromosomes.

Using the chromosome mapping of the PepBS satellite DNA, we detected sites that coincided with all the NORs revealed by the Ag-NOR method. Accordingly, we documented variation in the size and number of PepBS sites (Fig. 8). Moreover, we noted a difference in probe signal intensity, as the sites in chromosome 9 were much brighter than those in chromosomes 7 and 8 (Fig. 8).

**Figure 9:**
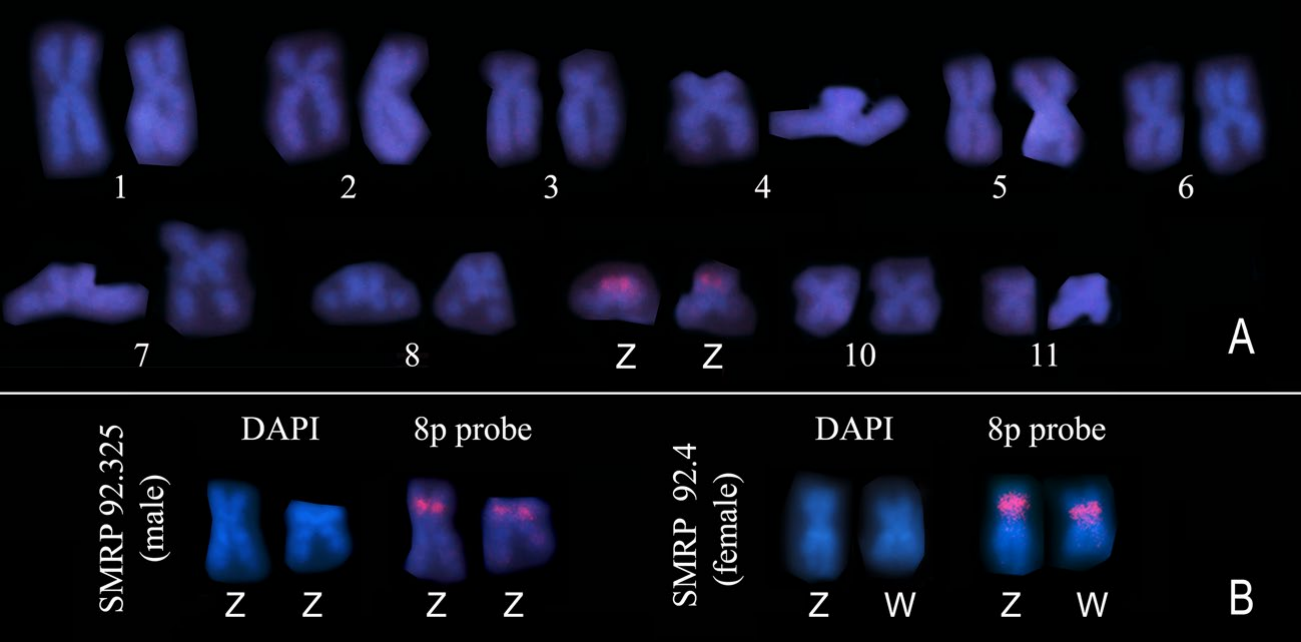
Mapping of the 8p probe in the sex chromosomes of specimens from the contact zone between *Physalaemus ephippifer* and L1. (**A**) Karyotype of a male specimen from SPAB (SMRP 92.325) hybridized to the 8p probe. (**B**) Sex chromosomes of a male and a female specimen from SPAB hybridized to the 8p probe.

Using in situ hybridization with the 8p probe, which previously detected the sex chromosomes of *P. ephippifer* and chromosome 9 of L1^29^, we documented a pericentromeric region of the short arm of both Z and W chromosomes of specimens from SPAB (Fig. 9). We observed size heteromorphism of this chromosomal region, but it was not related to sex (Fig. 9B).

### Cytogenetic analyses of specimens from Balsas

**Figure 10:**
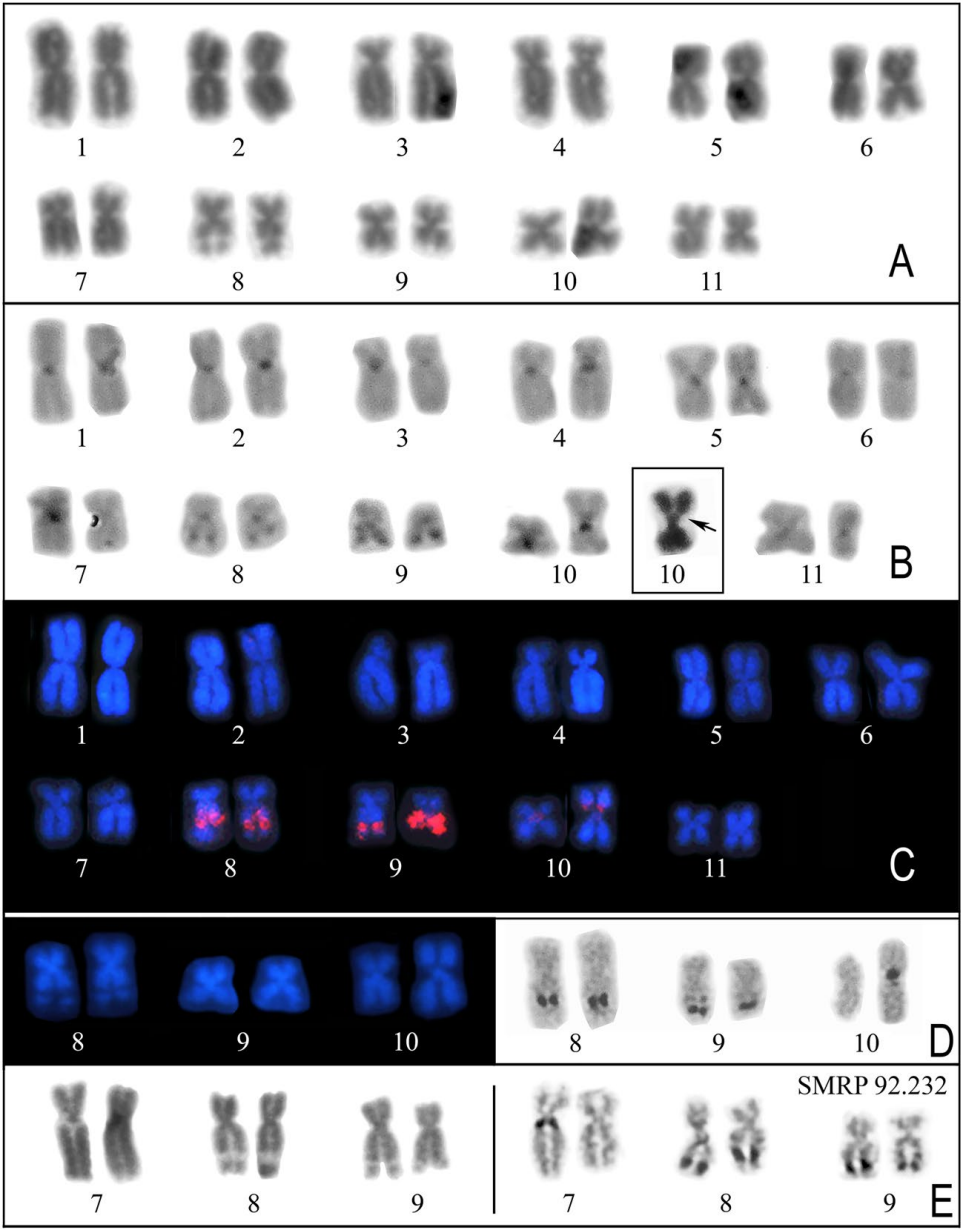
Karyotype of specimens from Bal. (**A**–**C**) Karyotype of the specimen SMRP 92.226 stained by Giemsa (**A**), C-banded (**B**), and hybridized to the PepBS probe (**C**). In the inset in B, the same NOR-bearing chromosome 10 shown in the karyogram is shown stained by Giemsa. Note that the pericentromeric C-band is in the long arm, while the NOR [seen as secondary constriction in the Giemsa-stained chromosome (arrow)] is in the short arm of chromosome 10. (**D**–**E**) NOR-bearing chromosomes of the specimens SMRP 92.226 (**D**) and SMRP 92.232 (**E**) stained by DAPI or Giemsa (on the left) and subjected to the Ag-NOR method (on the right). Note that the secondary constrictions in the DAPI and Giemsa-stained chromosomes coincide with NORs.

The three analyzed males had a 2n = 22 karyotype, which was composed of five pairs of metacentric chromosomes and six pairs of submetacentric chromosomes (Fig. 10). The Ag-NOR method revealed NORs in chromosomes 8 and 9 in all the specimens, with chromosome 9 being polymorphic in relation to the presence of an interstitial NOR besides a distal one (Fig. 10D,E). In addition, extra pericentromeric NORs were present in one homologue of pair 10 and one homologue of pair 7 in the specimens SMRP 92.226 and 92.232, respectively (Fig. 10D,E).

The hybridization of the PepBS probe to the karyotype of the SMRP 92.226 detected all the NORs, and the signal probe coincident with the NORs in chromosome 9 was brighter than those observed in chromosomes 8 and 10 (Fig. 10C, Supplementary Fig. S6). Although the NOR of chromosome 10 was similar in size to those of chromosomes 8 and 9, the probe signal coincident with this NOR was much smaller (Fig. 10C, Supplementary Fig. S6). The PepBS probe also revealed a small signal near the centromere of chromosome 10 which had no NOR detected by the Ag-NOR method (Fig. 10C, Supplementary Fig. S6).

All the detected NORs, especially those of chromosomes 7, 8, and 10, coincided with secondary constrictions in Giemsa and DAPI-stained metaphases (Fig. 10A,D,E) and the NORs in chromosome 9 also coincided with C-bands (Fig. 10B). In some metaphases, a faint C-band was noted adjacent to the NOR in chromosome 8. The C-banding also revealed the centromeric region of all chromosomes, an interstitial band in the short arm of chromosome 5, and a pericentromeric band in the long arm of chromosome pair 10 (Fig. 10B).

## Discussion

Together, our analyses of mtDNA, 3RAD, and cytogenetic data from the *P. cuvieri – P. ephippifer* species complex revealed evidence of admixture between *P. ephippifer* and L1, which suggests an important role for historic contact and introgression in structuring the current distribution of genetic diversity—and potentially the evolution of sex chromosomes—in this group. In addition, our results provide evidence of admixture between L1 and L3, raise questions about the relationship between L2 and L3, and expand our knowledge about the diversity within this species complex, leading to the recognition of two new lineages (i.e. L4 and VBST) that warrant further investigation. We discuss each of these topics in greater detail and highlight opportunities for additional research below.

### Admixture between *P*. *ephippifer* and L1: a potential driver of sex chromosome evolution

With respect to *P. ephippifer* and L1, we found some discordant results between the phylogenetic analyses based on mtDNA and 3RAD data. Cytonuclear incongruence is typically expected either in scenarios involving hybridization or incomplete lineage sorting^20,21,34^. However, our ABBA-BABA tests support hybridization as the most likely explanation for the patterns we observed. Accordingly, our findings indicate that the region encompassing São Pedro da Água Branca, Vila Nova dos Martírios, São Francisco do Brejão—Trecho Seco, and Imperatriz (the SPAB-VNM-TS-Imp region) may represent a contact zone between the sister lineages *P. ephippifer* and L1.

The frequency of mtDNA haplotypes, the topology of the 3RAD phylogeny and phylogenetic network, the results of Structure analyses and PCAs, and the cline analyses are all consistent with the greatest *P. ephippifer* ancestry in SPAB (located at the western side of the inferred contact zone) and the greatest L1 ancestry in Imp (located at the southeastern limit of this region). If evidence of gene flow from ABBA-BABA tests reflected limited but ongoing hybridization between individuals of the two parental lineages (i.e. *P. ephippifer* and L1), we would expect to find a continuum of ancestry proportions in each site—reflecting F1 hybrids and early-generation backcrosses. Instead, we found similar ancestry proportions in all individuals collected at each site, and no evidence of pure parental individuals of *P. ephippifer* or L1 in any locality within this contact zone. The very low interspecific heterozygosity in the samples from SPAB, VNM, TS, and Imp supports this inference, with no F1 hybrid identified in the 3RAD analyses. Our geographic cline analyses of both 3RAD and mtDNA data suggest a west-to-east change in ancestry in this region, but a denser geographic sampling is still necessary for a proper analysis of the extension of the contact zone between *P. ephippifer* and L1.

Our cytogenetic data also did not show evidence of ongoing hybridization between parental *P. ephippifer* and L1. Because the karyotypes of *P. ephippifer* and L1 can be easily distinguished from each other, particularly by the presence of heteromorphic sex chromosomes in *P. ephippifer*^26,27^, we would expect to easily recognize first-generation hybrids if they exist. The karyotypes of such hypothetical F1 hybrids would include, for example, a heteromorphic chromosome pair 8, composed of only one NOR-bearing homologue (inherited from L1), and a pair 9 composed of one chromosome 9 from L1 and one sex chromosome (Z or W) from *P. ephippifer*. Instead, our cytogenetic analyses did not reveal karyotypes formed by the simple combination of chromosomes currently found in the two parental lineages. On the contrary, our analysis detected unique characteristics of the specimens collected in this hybrid zone, including particular Z and W sex chromosomes, which karyotypically distinguish these specimens from both *P. ephippifer* and L1. Also, in the specimens from SPAB and VNM, chromosome pair 7 bears a NOR, which is a feature not found in any *P. ephippifer* or L1 specimens analyzed to date.

Therefore, our data show the cytogenetic distinctiveness of the specimens from SPAB and VNM. This finding is consistent with the PCA of 3RAD markers—which clearly separated the samples from the SPAB-VNM-TS-Imp region—as well as with the Structure analysis—which showed the strongest support for K = 3 when comparing 3RAD markers of *P. ephippifer*, L1, and specimens from the SPAB-VNM-TS-Imp region. Whether these populations are reproductively isolated (e.g. through genomic incompatibility or behavior) remains unclear and warrants further research.

The origin of new species through hybridization, a process known as hybrid speciation, has long been studied^18,35,36^. Change of ploidy^3^ and chromosomal rearrangements are known to play major roles^37^ in allopolyploid and homoploid speciation, respectively. Among anurans, the emergence of new species involving polyploid lineages has been more commonly reported (e.g. Refs.^38–40^), but there is also evidence of hybrid speciation unrelated to polyploid species. An interesting case was reported for *Litoria genimaculata* (currently *Ranoidea genimaculata*^41^), which exhibited two divergent lineages that hybridized in two distinct contact zones^42^. In one of them, significant premating isolation of the hybrid lineage was found^42^, supporting the description of this lineage as a new species^43^. Another intriguing case refers to the ranid frog *Glandirana rugosa*, in which new lineages have emerged from the contact and hybridization of groups with diverging sex chromosomes^44–48^.

**Figure 11:**
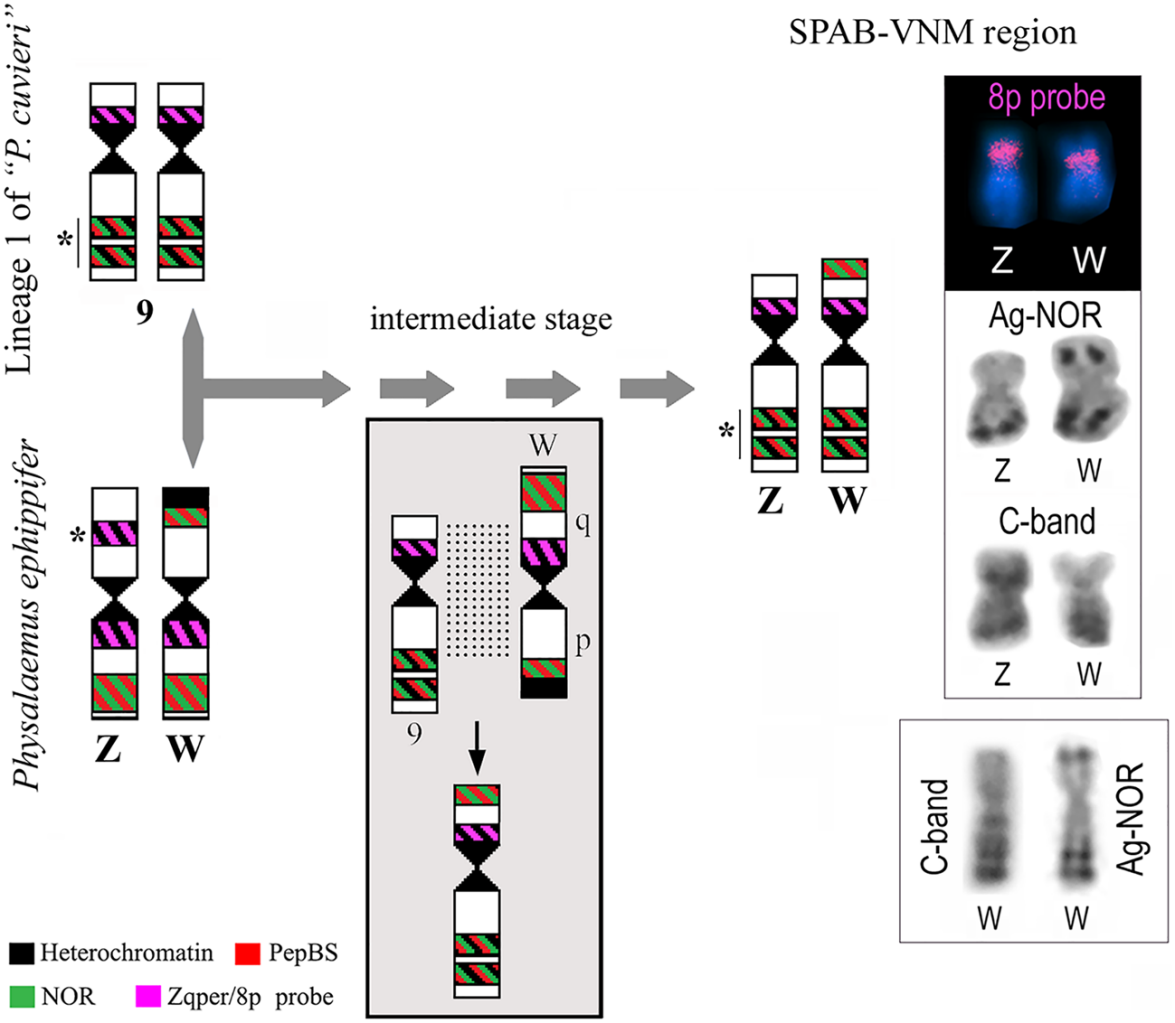
Hypothetical evolution of sex chromosomes promoted by hybridization between *Physalaemus ephippifer* and L1. The W chromosome currently found in specimens from SPAB-VNM may result from recombination between chromosome 9 of L1 and the W chromosome of *P. ephippifer*, as illustrated in the gray box. Dotted lines indicate the supposed homologous region between chromosome 9 of L1 and the W chromosome of *P. ephippifer*. Note that the W chromosome found in specimens from SPAB-VNM bears, in the long arm, NORs that coincide with C-bands (as chromosome 9 of L1) and in the short arm, a NOR that does not colocalize with C-bands (as the W chromosome of *P. ephippifer*).

A detailed analysis of the sex chromosomes found in specimens from SPAB and VNM shows that their W chromosome is similar to that of *P. ephippifer*^26^ in having NOR in the short arm but differs in not having an evident terminal heterochromatic block in the short arm, adjacent to the NOR (Fig. 11). However, the long arm of the W chromosome in specimens from SPAB and VNM has NORs detectable by C-banding (Fig. 11), differentiating it from the long arm of the W chromosome of *P. ephippifer*, which bears NORs not coincident with C-bands^26^. Finally, the 8p probe detected a pericentromeric region in the short arm of the W chromosome of specimens from SPAB and VNM, while it mapped pericentromerically to the long arm of the W chromosome of *P. ephippifer*^26^ (Fig. 11).

The Z chromosome of specimens from the contact zone is more similar to chromosome 9 of L1 than to the Z chromosome of *P. ephippifer* (Fig. 11). The Z chromosome of specimens from SPAB and VNM and chromosome 9 of L1^27,29^ have a pericentromeric C-band in the short arm, which coincides with the region detected by the 8p probe, and NORs in the long arm, which coincides with C-bands and PepBS sites. Another similarity between the karyotype of L1 and that found in SPAB and VNM is the presence of NOR-bearing chromosomes 8.

Based on the abovementioned comparative analysis of the sex chromosomes of *P. ephippifer* and their homeologous chromosome 9 of L1, the inferred scenario of admixture between *P. ephippifer* and L1 makes it highly plausible that these chromosomes have undergone recombination in the past. Given their similarity, it is reasonable to posit that 9p of L1 is homologous to Wq of *P. ephippifer*, and 9q of L1 to Wp of *P. ephippifer* (Fig. 11). Consequently, we hypothesize that the W chromosome from SPAB-VNM may have resulted from recombination between chromosome 9 of L1 and the W chromosome of *P. ephippifer* (Fig. 11). Hypothetical recombination between these chromosomes would result in a chromosome with (1) a pericentromeric block in the short arm detected by the 8p probe (as observed in 9p of L1 and Wq of *P. ephippifer*), (2) a distal NOR in the short arm that does not colocalize nor is adjacent to C-band (a condition also observed in Wq of *P. ephippifer*), and (3) NORs in the long arm that coincide with C-bands (as observed in 9q of L1) (Fig. 11). Under this scenario, admixture between *P. ephippifer* and L1 would have promoted the origin of a new sex chromosome system, composed of Z and W chromosomes with a lower level of differentiation than that observed between the sex chromosomes of *P. ephippifer*. Thus, gene flow (or interspecific hybridization) may have been an important driver of karyotypic evolution in this group of frogs (see further discussion below).

However, we cannot disregard alternative hypotheses in which the Z and W chromosomes currently found in SPAB and VNM might have arisen from multiple mutations/rearrangements occurring on ancestral chromosomes. Additional genomic studies would be valuable for identifying linkage groups within the W chromosomes of SPAB-VNM and *P. ephippifer* and chromosome 9 of L1 to further test these hypotheses. Additionally, the full extent of the contact zone, as well as the timing and duration of gene flow, should be addressed in future studies, which should include a denser geographic sampling—also comprising specimens from beyond the SPAB-VNM-TS-Imp region—and acoustic and morphological data. These data will also allow us to evaluate whether the populations in SPAB-VNM-TS-Imp represent a narrow hybrid zone facilitating ongoing (but limited) gene flow between *P. ephippifer* and L1 or these admixed populations are reproductively isolated from parental lineages.

### Gene flow between L1 and L3 and between L2 and L3

In addition to the contact between *P. ephippifer* and L1, we found preliminary evidence of gene flow between two non-sister lineages—L1 and L3. In a previous study, delimitation tests based on mtDNA strongly supported L3 as a valid species, distinct from L1^25^. However, in our analyses, from the ten specimens from Bal included in our mtDNA analyses, six had mitochondrial haplotypes that clustered within L1 and four had haplotypes that clustered within L3. Although all six specimens from Bal included in both the 3RAD and mtDNA analyses had mitochondrial haplotypes from L1, the topology of the 3RAD phylogeny and phylogenetic network and the results of the Structure analyses and PCA were all consistent with introgression from L3 into L1. Additionally, the ABBA-BABA test resulted in a D-statistic that was significantly negative, thus supporting the hypothesis of introgression as an explanation for the discordant inferences from mtDNA and 3RAD markers.

The cytogenetic analysis of specimens from Bal showed additional NORs in chromosomes 7 and 10. L3 is highly polymorphic regarding NOR location^27^ and NOR-bearing chromosomes 7 and 10 similar to those found in Bal were found in L3 (Supplementary File S1). Accordingly, we hypothesize that the NOR-bearing chromosomes 7 and 10 found in Bal have introgressed from L3. Therefore, the NOR polymorphism observed in Bal would have arisen from introgressive hybridization (see further discussion below). However, our small sample size and the limited geographic extent of our samples prevent us from drawing stronger conclusions about the timing or frequency of gene flow.

Finally, we found some preliminary evidence for gene flow between L2 and L3 in the discordance between the phylogenetic placement of a specimen from Pirenópolis (SMRP 92.307) in the mtDNA and 3RAD phylogenies. In the mtDNA phylogeny, we recovered this sample within L2 (Fig. 2), but it was sister to L3 in the 3RAD phylogeny (Fig. 3) and clustered separately with one sample from Paranã (SMRP 92.308) in the 3RAD phylogenetic network (Supplementary Fig. S4).

While secondary contact and gene flow between pairs of anuran species or lineages have been increasingly reported^49–53^, it is worth noting that admixture involving multiple lineages has also been documented^54–56^. When multiple divergent lineages come into contact, the extent of hybrid zones and the degree of admixture may vary among distinct contact zones. Therefore, comparative analyses of multiple contact zones can provide valuable insights into the evolutionary processes involved in species formation. For instance, Wen & Fu^55^’s study on the frog *Odorrana margaretae* revealed that one contact zone exhibited extensive admixture, whereas another zone occupied a narrow geographic area and showed limited admixture of parental lineages. In the case of the *P. cuvieri – P. ephippifer* species complex, the historical hybridization inferred between *P. ephippifer* and L1 potentially contributed to the emergence of a new karyotype (as discussed above). Conversely, we lack evidence indicating a similar impact from the inferred contact between L1 and L3 on karyotype divergence. Further characterization of each contact zone, including analyses of the direction, timing, and frequency of hybridization events, remains essential for a comprehensive study of this species complex. Such investigations may offer unique insights into the speciation process.

### Additional genetic diversity unveiled in the *P*.* cuvieri* – *P*. *ephippifer* species complex

Our expanded geographic sampling also clarified some general aspects of the distribution of genetic diversity within the *P. cuvieri – P. ephippifer* species complex and raises new questions that warrant further study. For example, we demonstrated that L3, which was previously restricted to Porto Nacional^25,57^, also occurs in the region of Paranã.

In contrast, our analyses showed that the specimens from VBST, also located in Central Brazil, differ from those of Porto Nacional and Paranã. The mtDNA phylogeny suggested that VBST is sister to L3—a relationship that was not recovered in the phylogenetic analysis of the 3RAD dataset. In all the species delimitation tests reported previously^25^, L3 was recovered as a valid species. Here, we found approximately 3% of nucleotide divergence between VBST and L3 in the 16Sar–16Sbr fragment, a threshold value proposed by Fouquet et al.^58^ and Lyra et al.^59^ to flag candidate species of neotropical frogs. Therefore, further analyses are of fundamental importance to evaluate the taxonomic status of the VBST clade.

Finally, we recovered a clade composed of samples from Marabá and Parauapebas in the phylogenetic analyses of mtDNA and 3RAD data, which we refer to as Lineage 4 (L4). All analyses supported the close relationship between L4, L1, and *P. ephippifer*, but the precise phylogenetic relationships within this clade remain to be elucidated.

These findings and the abovementioned evidence of multiple contact zones reinforce the need for a comprehensive taxonomic revision of the *P. cuvieri – P. ephippifer* species complex. As anticipated^25^, it will be a challenging task due to formal issues regarding the original species descriptions—including the imprecise information on the type-locality for *P. cuvieri* Fitzinger 1826 and the fact that its type specimens are not included in recent type lists^41^—but also because it will need comprehensive morphological and acoustic analyses complementing genetic studies in an integrative framework.

### Hybridization as a driver of chromosome evolution in anurans

Interspecific hybridization plays an important role in shaping karyotype diversity^37,60^. In scenarios where the hybrids are viable and fertile, backcrosses may lead to the introgression of chromosomal variants from one species to another, which may result in stable chromosomal polymorphisms^61^. In contrast, when hybrid incompatibility promotes an intrinsic postzygotic reproductive barrier (which might be complete or incomplete, depending on the severity of hybrid incompatibility), introgression is prevented and natural selection favors interspecific divergence, intensifying the reproductive isolation through reinforcement^5,6^. One interesting category of hybrid incompatibility is related to the presence of distinct sex chromosomes in the parental species, underscoring the importance of sex chromosomes in speciation^30–33^. In this scenario, interspecific differences in sex chromosomes may accumulate following reproductive isolation, but new sex chromosome systems might arise as a consequence of hybridization^45,47,48^.

In the species complex studied here, we found evidence supporting the involvement of natural hybridization between divergent lineages in two distinct phenomena: (1) the polymorphism of NORs (since the analyses of specimens from Bal suggested that NOR-bearing chromosomes from L3 were introgressed into L1); and (2) the origin of a new sex chromosome system (found in SPAB and VNM).

To date, the involvement of interpopulational hybridization in the origin of new sex chromosome systems in anurans had only been reported for *Glandirana rugosa*^44–48^. In this extraordinary case, six major geographic groups are recognized: two with male heterogamety and homomorphic sex chromosomes (the West-Japan and East-Japan groups, which are supposed to be original groups); two with female heterogamety and heteromorphic sex chromosomes (the ZW and Neo-ZW groups); one with male heterogamety and heteromorphic sex chromosomes (the XY group); and one with male heterogamety and homomorphic sex chromosomes (the Neo-West-Japan group)^45,46,48^. All these distinct sex chromosome systems would have emerged after hybridization between groups, with at least four hybrid zones where divergent groups would have secondarily contacted and hybridized^45,47,48^. During the evolution of these sex chromosome systems, a subtelocentric chromosome originated from a metacentric one (which gave rise to a heteromorphic condition) losses of W and Y chromosomes supposedly occurred in some of the contact zones, and new female-determining gene(s) emerged, promoting parallel transitions from male to female heterogamety^45^.

Cytogenetic analyses showed no differences between the karyotype found in the West-Japan group and that of the Neo-West-Japan group of *Glandirana rugosa*, two lineages that supposedly evolved from the hybridization between the West-Japan group and either the Neo-ZW or the XY group (both groups with heteromorphic sex chromosomes)^48^. Based on such evidence, Ogata et al.^48^ argued that the loss of W or Y chromosomes may have been involved in the process that secondarily originated a homomorphic sex chromosome system. Because molecular comparisons of the sex chromosomes of the West-Japan and Neo-West-Japan groups (including their sex-determining locus) remain to be done, whether the condition found in the Neo-West-Japan group is exactly a reversion to the original state or not is still an open question^48^. In either case, hybridization is involved in the origin of homomorphic sex chromosomes from a heteromorphic system, which led Ogata and colleagues to advocate that it is a mechanism that contributes to the prevalence of homomorphic sex chromosomes in anurans.

Here, we suggest that hybridization may have also played an important role in the origin of new sex chromosomes in the *P. cuvieri – P. ephippifer* species complex. The new ZZ/ZW sex chromosome system we found in specimens from SPAB and VNM shows a lower level of chromosome heteromorphism than found in *P. ephippifer*. We hypothesize that such a decrease in heteromorphism results from the recombination between chromosome 9 of L1 and chromosome W of *P. ephippifer*, after these lineages secondarily contacted and hybridized (Fig. 11). In this scenario, hybridization would be important to counteract sex chromosome differentiation, playing a role similar to that attributed to the male-to-female sex reversal in XX/XY systems in the fountain-of-youth model proposed by Perrin^62^. According to Perrin’s model, the occurrence of sex-reversed XY females in amphibians would increase the recombination between X and Y chromosomes, preventing sex chromosome differentiation and thus contributing to the prevalence of homomorphic sex chromosomes in these vertebrates. In this model, deleterious mutations accumulated in “old” Y chromosomes are lost by recombination, giving rise to “younger” Y chromosomes^62^. Therefore, we agree with Ogata et al.^48^ in proposing hybridization as an important force against sex chromosome differentiation and we suggest that hybridization could be considered an additional mechanism promoting recombination and rejuvenating sex chromosomes. In this sense, sex reversal in XX/XY systems and hybridization would be both considered fountains of youth.

## Conclusion

We found evidence of admixture in multiple contact zones in the *P. cuvieri – P. ephippifer* species complex, suggesting that hybridization has promoted rapid karyotypic evolution in this group, contributing to NOR polymorphism and the emergence of a new sex chromosome system. Our results support previous hypotheses that hybridization may contribute to counter sex chromosome differentiation in anurans.

## Methods

We studied an extended sample comprising five lineages of the *P. cuvieri – P. ephippifer* species complex, including specimens from a geographical area situated between previously documented localities of *P. ephippifer*, and lineages L1 and L3 of *P. cuvieri* sensu lato (Fig. 1). We generated mitochondrial sequences, 3RAD markers, and cytogenetic data from sampled specimens and combined these data with earlier datasets. Supplementary Table S1 lists the specimens used in each analysis and identifies new samples in this study. Details regarding each procedure and analysis are presented below.

### Specimen and tissue collection

We collected new specimens of *P. ephippifer* (n = 9) and four lineages historically classified as *P. cuvieri*. Using informal lineage names as described in Lourenço et al.^57^, these four lineages are: Lineage 1A (L1A; n = 1), Lineage 1B (L1B; n = 17), Lineage 2 (L2; n = 4), and Lineage 3 (L3; n = 4) (Supplementary Table S1). We also collected 37 specimens from sites located between the known geographical distribution boundaries of *P. ephippifer*, L1, and L3, which included the municipalities of Imperatriz (Imp; n = 2), São Francisco do Brejão—Trecho Seco (TS; n = 5), Vila Nova dos Martírios (VNM; n = 4), São Pedro da Água Branca (SPAB; n = 21), Marabá (Mar; n = 1), and Parauapebas (Par; n = 4) (Fig. 1; Supplementary Table S1). We also sampled the municipality of Vila Bela da Santíssima Trindade (VBST) and included additional samples from Balsas (Bal)—a region located at the western limit of the known distribution area of L1 (Fig. 1; Supplementary Table S1) and formerly analyzed by Lourenço et al.^57^ and Nascimento et al.^25^. As an outgroup for 3RAD analyses, we also included specimens of *P. centralis* (Supplementary Table S1).

We collected all specimens under a permit issued by the Chico Mendes Institute for Biodiversity Conservation/Biodiversity Authorization and Information System (ICMBio/SISBIO) (process #32483). We anaesthetized all frogs using 2% lidocaine and dissected liver samples for DNA analyses and intestine samples for cytogenetic analysis. We obtained genomic DNA from liver samples following the protocol described by Medeiros et al.^63^. All experiments were performed in accordance with relevant guidelines and regulations and approved by the Committee for Ethics in Animal Use of the University of Campinas (CEUA/UNICAMP) (permit number 4802-1/2018). We deposited all specimens in the amphibian collection of the Zoology Museum “Prof. Adão José Cardoso” at the University of Campinas (ZUEC) or in the herpetological collection of the Federal University of Maranhão (HUFMA). When pertinent, this study was performed in compliance with the ARRIVE guidelines.

### Mitochondrial DNA analyses

We generated mitochondrial DNA (mtDNA) sequence data, including the tRNA-Val gene and partial sequences of 12S and 16S rRNA genes, for 66 of the specimens collected. We isolated and amplified fragments of interest by PCR using the primer pairs MVZ59^64^ – Titus I^65^ and 12L13^66^ – 16Sbr^67^. We purified PCR products using a Wizard SV Gel and PCR Clean-up System (Promega) and sequenced amplicons using a BigDye Terminator kit (Applied Biosystems) in an ABI 3730xL DNA Analyzer automatic sequencer (Applied Biosystems). We conducted sequencing reactions with the aforementioned primers and also with primers MVZ50^64^, 16SL2a^68^, 16H10^68^, and 16Sar^67^. We edited the resulting nucleotide sequences using CodonCode Aligner software v.5.1.5 (www.codoncode.com) or BioEdit Sequence Alignment Editor software v.7.2.5^69^, yielding concatenated segments delimited by primers MVZ59 and 16Sbr. All the obtained sequences were deposited in GenBank (see Supplementary Table S1 for accession numbers).

We aligned the newly generated sequences with those available on GenBank to create a dataset with mitochondrial sequences from 123 specimens from the *P. cuvieri – P. ephippifer* species complex (Supplementary Table S1). We also included sequences of representatives from the remaining eight species currently assigned to the *P. cuvieri* species group (i.e. *P. albifrons*, *P. albonotatus*, *P. atim*, *P. centralis*, *P. cuqui*, *P. erikae*, *P. fischeri*, and *P. kroyeri*), as well as sequences from representatives of each of the four other species groups in the *P. cuvieri* clade, and sequences of *P. nattereri*—a species of the *P. signifer* clade (which is the sister clade of the *P. cuvieri* clade)^57^—in our data matrix (Supplementary Table S1). We aligned all sequences with MAFFT v.7^70^ (https://mafft.cbrc.jp/alignment/server/), using the G-INS-i option, resulting in a data matrix composed of 153 samples and 2324 characters.

We estimated a maximum-likelihood phylogeny from the mtDNA dataset using RAxML^71^ as implemented in the CIPRES Science Gateway v.3.3^72^, employing the GTRCAT nucleotide substitution model. We assessed node support with a bootstrap algorithm, using different random seeds and 1000 replicates. We used *P. nattereri* as the outgroup to root the inferred phylogeny.

We estimated genetic distances between and within major lineages in the *P. cuvieri – P. ephippifer* species complex using MEGA v.7.0^73^, treating alignment gaps and missing data as pairwise deletions. We calculated uncorrected genetic distances (*p*-distances) from the 12SrRNA-tRNA-val-16SrRNA fragment and also from the partial segment of 16S rRNA gene flanked by the primers 16Sar and 16Sbr, which is commonly used for evaluating interspecific variation in frogs^58,59^.

We obtained a list of 68 distinct mtDNA haplotypes related to the *P. cuvieri-P. ephippifer* species complex using DNAsp v.5.10.01^74^ and constructed a haplotype network using the median-joining method (MJN) in the software Network v.2.0.1.1^75^. We ignored sites with missing data and alignment gaps in this analysis. Due to sequencing failure at the 3’ end of the 12S rRNA gene, specimens SMRP 92.228, SMRP 92.229, SMRP 92.242, SMRP 92.306, SMRP 92.307, and PS555 were excluded from this analysis, as they had 235 to 334 bp of missing data, leading to the loss of a common 151 bp segment.

### 3RAD library preparation, sequencing, and assembly

We generated genome-wide SNP data for 81 specimens of the *P. cuvieri – P. ephippifer* species complex and two specimens of *P. centralis* (Supplementary Table S1) using the 3RAD (triple-digest RADseq) protocol described by Bayona-Vásquez et al.^76^. In brief, we digested DNA samples from 57 specimens with the enzymes MspI, ClaI, and BamHI-HF, ligated the resulting DNA fragments to iTru adapters specific to MspI and BamHI-HF cutsites, and cleaned ligation products with SpeedBeads. We then conducted individual PCRs with iTru5 and iTru7 primers^77^ for these 57 ligation products plus 26 ligation products previously generated by Nascimento et al.^25^ from samples of the *P. cuvieri – P. ephippifer* species complex.

We normalized and pooled PCR products and size-selected this pool for 450–550 bp on a Pippin Prep. Finally, we combined these pooled, size-selected libraries for sequencing on an Illumina NovaSeq, targeting approximately 10 million paired-end, 150-basepair reads (PE 150) per sample.

We quality-filtered and assembled reads using ipyrad v.0.9.84^78,79^. We trimmed forward and reverse reads to 130 bp. We then used a clustering threshold of 0.85, used a minimum depth of six reads per locus, and required that a locus be present in at least four samples to be included in the final assembly. Raw sequence reads are available from the NCBI SRA (PRJNA997238).

### 3RAD data analyses

First, we inferred phylogenetic relationships from the full 3RAD dataset (n = 83 individuals, n = 2,757,035 SNPs) and the .u.snps.phy output file from ipyrad (i.e. using only one random SNP per locus; n = 83 individuals, n = 205,030 SNPs) using RAxML v.8.2^80^ and RAxML^71^ in the CIPRES Science Gateway v.3.3^72^, respectively. We used the GTRCAT model in both analyses and assessed node support with a bootstrap algorithm, using different random seeds and 100 replicates of the .u.snps.phy dataset. We used the outgroup *P. centralis* to root the inferred phylogeny.

Second, we inferred a distance-based phylogenetic network using Hamming distances and the NeighborNet algorithm^81^ as implemented in package phangorn v.2.11.1^82,83^ in R v4.1.0^84^. We also conducted this analysis using one random SNP per locus (n = 83 individuals, n = 205,030 SNPs).

Third, we conducted a series of principal component analyses (PCAs) using the ipyrad-analysis toolkit in ipyrad v.0.9.84^78,79^. For each analysis, we: (1) made “population maps” assigning each sample to groups defined by lineage (e.g. L1) or sampling site (e.g. VNM); (2) included only loci with data in at least 20% of individual samples within each group and in at least 50% of overall samples; (3) included all SNPs with a minor allele frequency (MAF) ≥ 0.05; and (4) imputed missing data using kmeans clustering, with k = 10. We conducted three separate PCAs. In the first PCA, we included all samples from our assembled dataset (n = 83 individuals, n = 20,224 SNPs). In the second PCA, we investigated potential evidence of gene flow between *P. ephippifer* and L1 by including only samples from these lineages and their putative contact zone (i.e. SPAB, VNM, TS, and Imp) (n = 53 individuals, n = 45,800 SNPs). In the third PCA, we evaluated potential evidence of gene flow between L1 and L3 by including only samples from these two lineages, plus samples of *P. ephippifer*—the closest relative of L1 (n = 27 individuals, n = 14,782 SNPs)*.* For each analysis, we plotted the first two PC axes, jointly representing 20–38% variance in each dataset.

Fourth, we used the Bayesian population clustering program Structure v2.3.4^85^ as implemented in the ipyrad-analysis toolkit in ipyrad v.0.9.84 to explore patterns of genetic structure and admixture^78,79^. We conducted two separate Structure analyses. In the first analysis, we assessed gene flow between *P. ephippifer* and L1 by including samples from these two lineages and their putative contact zone (i.e. SPAB, VNM, TS, and Imp). Previous studies have demonstrated that Structure analyses can be sensitive to missing data^86^ and that biological signal can be masked by the inclusion of singletons^87^. Thus, we filtered our SNPs to include: (1) only loci with data in at least 20% of individual samples within each group and in at least 80% of overall samples; and (2) only loci with a MAF ≥ 0.02 (to exclude singletons). This dataset consisted of 53 individuals and 3465 SNPs after filtering. We conducted 10 replicate runs of 300,000 MCMC steps (with 30,000 burnin steps) for K = 1–7 and visually evaluated relative support for the optimal number of clusters by plotting ΔK^88^. In the second analysis, we focused on evaluating gene flow between L1 and L3 by including only samples from those two lineages (n = 17 individuals, n = 1,772 SNPs). We conducted this analysis as described above, except that: (1) we included only SNPs with a MAF ≥ 0.04 (to exclude singletons, accounting for a smaller number of individuals in the dataset); and (2) we evaluated only K = 1–5.

Fifth, we conducted a series of targeted ABBA-BABA tests^89,90^ to provide independent assessments of introgression, again using the ipyrad-analysis toolkit in ipyrad v.0.9.84^78,88^. We conducted five analyses, four of which were designed to evaluate the relative contribution of *P. ephippifer* and L1 to the ancestry of samples from each site in the putative contact zone (i.e. SPAB, VNM, TS, and Imp). The fifth analysis was designed to evaluate evidence for introgression of alleles from L3 into samples from Bal relative to other sites from L1. For each analysis, we used samples of *P. centralis* as an outgroup, started with our full SNP dataset (n = 2,757,035 SNPs), and evaluated support for mean estimates of d-statistics by plotting the distributions from 1000 bootstrap replicates in R v.4.1.0^84^. In each analysis, we conducted pooled tests in which we used all samples from a site and also separate tests for each constituent sample. For the first four analyses, the number of SNPs used in each test (i.e. the SNPs that fit either an ABBA or BABA pattern of allele sharing) varied from 2364–7575 for SPAB, from 3174–5923 for VNM, from 1907–3454 for TS, and from 2194–3114 for Imp; for the fifth analysis, the number of SNPs varied from 2146–4454. We plotted bootstrap replicates from both individual and pooled tests, and used Z-scores to calculate p-values and evaluate statistical significance.

### Cline analysis of the putative contact zone between *P*. *ephippifer* and L1

To further characterize the putative contact zone between *P. ephippifer* and L1 we used a series of cline analyses. Using the packages vcfR v1.14.0^91^ and SNPfiltR v1.0.1^92^ in R v4.3.1^84^, we filtered the full SNP dataset to include only samples from *P. ephippifer* and L1 (excluding the samples from Bal) and their putative contact zone (i.e. including SPAB, VNM, TS, and Imp) and meeting the following criteria (1) only biallelic SNPs; (2) only the first SNP per locus; (3) only SNPs with a minor allele count of ≥ 2; and (4) with ≤ 50% missing data (n = 46 individuals, n = 6815 SNPs). We then used code from^93^ to identify SNPs with fixed differences between *P. ephippifer* and L1 (n = 406 SNPs), and we used the package introgress v1.2.3^94^ to estimate hybrid indexes and calculate interspecific heterozygosity, which we visualized through a triangle plot. Next, we created geographic clines separately from both these hybrid indexes and from mtDNA haplotype frequencies. First, we transformed our two-dimensional geographic coordinate data (i.e. the sampling site locations) into a linear scale. We did this by fitting a line between the westernmost sampling site for *P. ephippifer* (Belém) and the easternmost sampling site for L1 (Araruna) in this dataset, calculating the point at which a line drawn perpendicular from each other site would intersect this line, and using the package geosphere v1.5–18^95^ to calculate the linear distance between the westernmost sampling site (Belém) and these points. We then used the package hzar v0.2–9^96^ to fit one geographic cline model using the hybrid indexes calculated in introgress and one using the mtDNA haplotype frequencies. In both cases, we estimated two parameters: the cline center and cline width. We fit each model using 1,000,000 MCMC steps with 1000 burnin steps.

### Cytogenetic analyses

To assess karyotypic variation in the contact zone between *P. ephippifer* and L1, we conducted cytogenetic analyses of seventeen individuals from SPAB and four from VNM (Supplementary Table S1). We also included three specimens from Bal—the site with evidence of admixture between L1 and L3 (see “Results”). For full specimen details, see Supplementary Table S1.

For chromosomal analyses, we injected frogs intraperitoneally with 2% colchicine (0.02 mL/g body weight). After four hours, we euthanized them with a cutaneous administration of 2% lidocaine (an overdose of 50 mg/g body) and removed the intestines (protocol approved by CEUA/UNICAMP, permit number 4802–1/2018). We then made chromosome preparations following the methods described in King and Rofe^97^, with modifications described in Gatto et al.^98^. The preparations are deposited in the cytogenetic collection “Shirlei Maria Recco Pimentel” (SMRP, Universidade Estadual de Campinas, Brasil). We also used some chromosome preparations previously described by Quinderé et al.^27^.

Next, we sequentially subjected these chromosome preparations to 10% Giemsa staining, C-banding^71^, and silver impregnation using the Ag-NOR method^99^. We also stained some C-banded metaphases by DAPI (0.5 µg/mL) after removing the Giemsa stain in 50% acetic acid. Additionally, we used fluorescence in situ hybridization (FISH) to map the satellite DNA PepBS^29^. We labeled a cloned PepBS fragment, previously isolated from *P. ephippifer* and available from the SMRP cytogenetic collection, with digoxigenin-dUTP (Roche) by PCR with primers PepBS-F and PepBS-R^29^ and used it as probe in FISH assays.

For cytogenetic analyses of specimens from the secondary contact zone between *P. ephippifer* and L1, we also used FISH to map the segment Zqper/8p using a probe originally constructed by Gatto et al.^29^ from chromosome microdissection, which was available from the SMRP cytogenetic collection. This probe (named 8p probe) is specific to the pericentromeric region of the long arm of Z chromosome of *P. ephippifer*, the pericentromeric region of the short arm of chromosome 9 of individuals from L1, and an interstitial region of the short arm of chromosome 8 of *Physalaemus* sp. (Western Pará and Viruá clades)^29^.

In all FISH assays, the hybridization step followed the method of Viegas-Péquignot^100^. We detected probes by anti-digoxigenin conjugated to rhodamine (Roche) (0.06 µg/mL) and stained chromosomes with DAPI (0.5 µg/mL).

### Supplementary Information

Supplementary Figures.

Supplementary Information 1.

Supplementary Table S1.

Supplementary Table S2.

## Data Availability

Newly obtained sequences were deposited in GenBank (OR005428-OR005495; OR078427-OR078428). Raw sequence reads are available from the NCBI SRA (PRJNA997238).
