# Supplementary material for: Multiple contact zones and karyotypic evolution in a neotropical frog species complex
Source: Sci Rep. 2024 Jan 11;14:1119. doi: 10.1038/s41598-024-51421-z (PMC10784582; doi:10.1038/s41598-024-51421-z)
Supplement: Supplementary file 1 — Supplementary Figures. [file 41598_2024_51421_MOESM1_ESM.pdf]

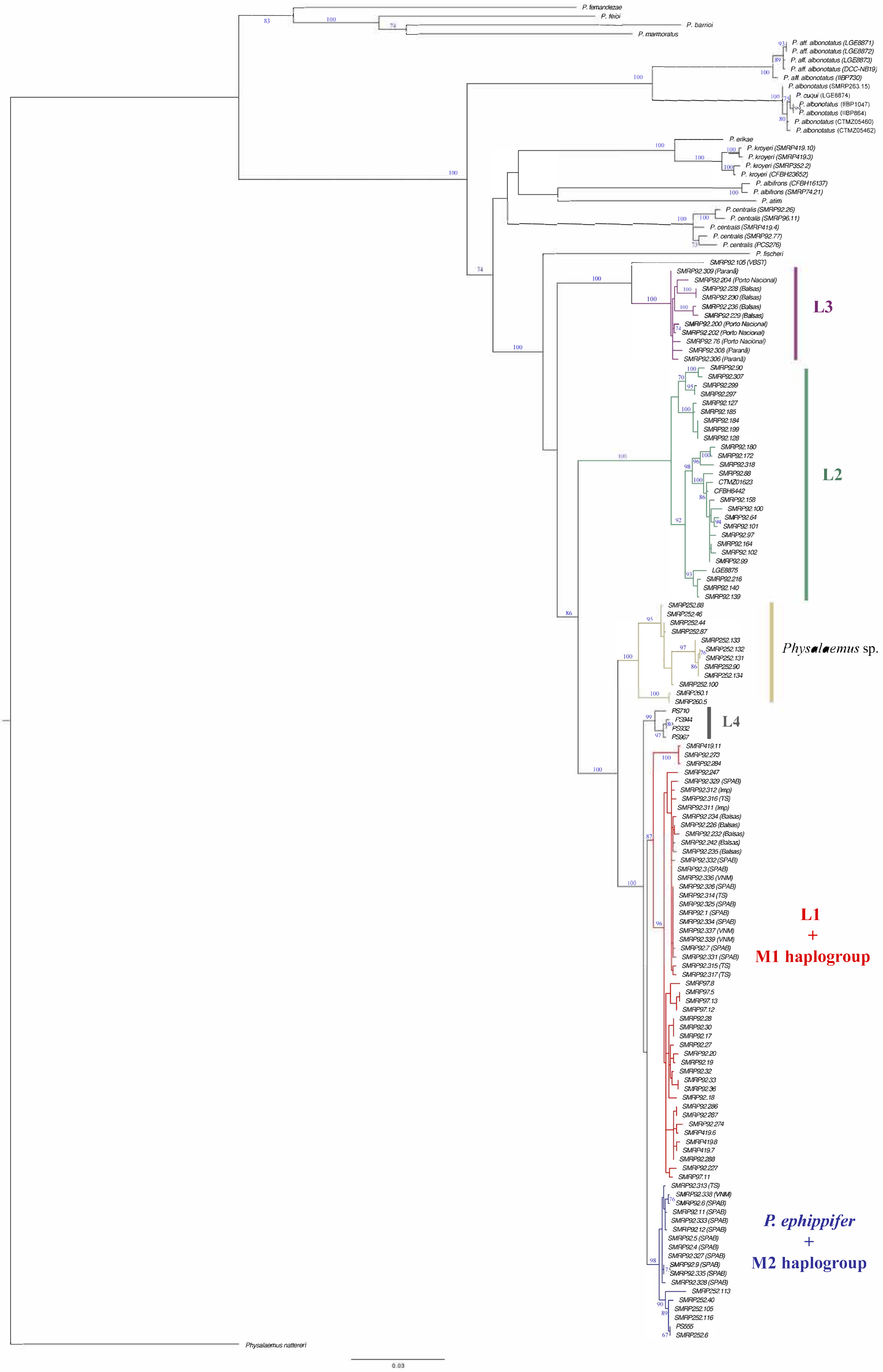

Supplementary Figure S1. Phylogenetic relationships inferred by RAxML analysis of mitochondrial DNA sequences. Numbers on the branches represent bootstrap values. (This figure is provided in high resolution. Utilize sequential zooms for detailed examination.)

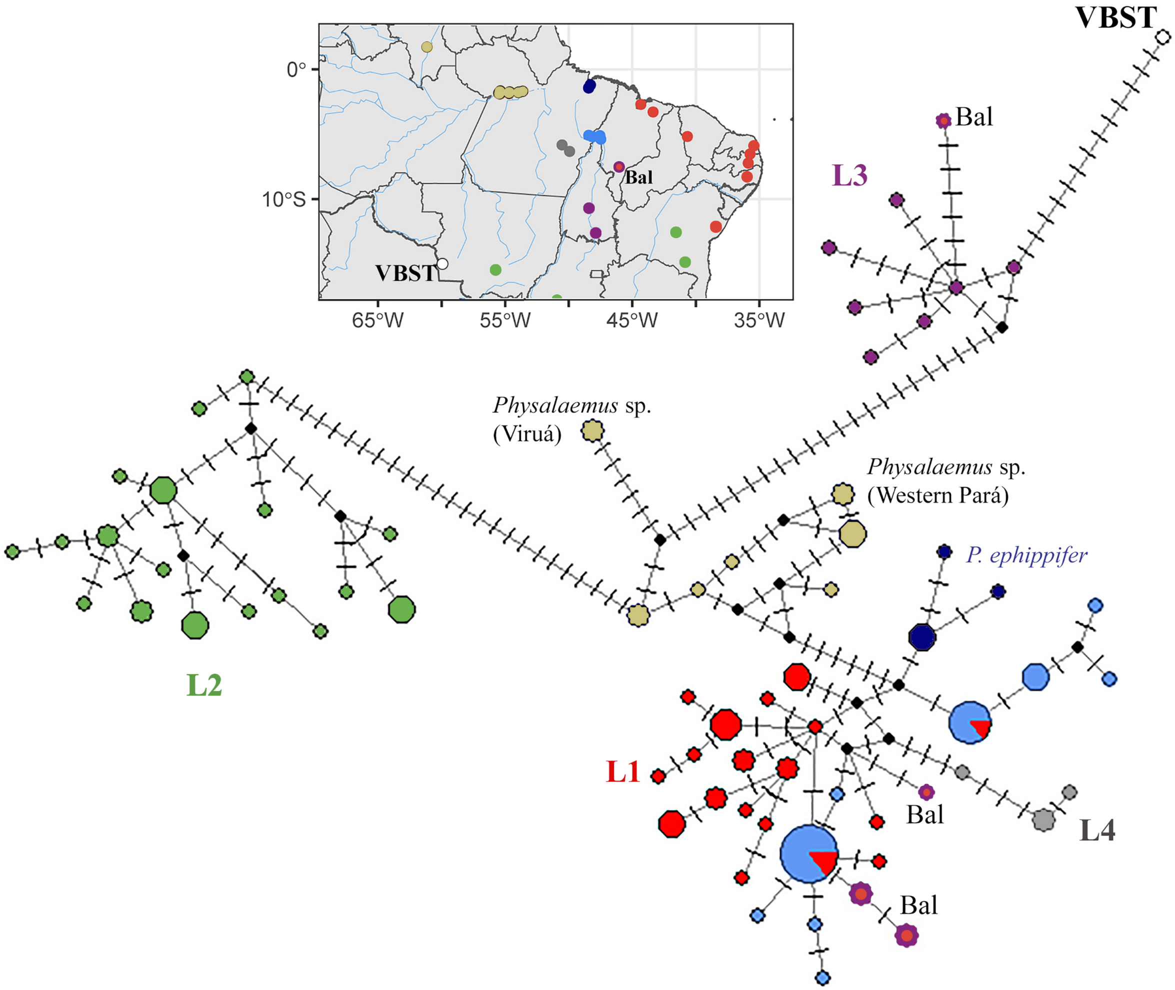

Supplementary Figure S2. Haplotype network inferred from MVZ59-16Sbr mitochondrial DNA sequences of specimens from the Physalaemus cuvieri – P. ephippifer species complex. Each circle represents a single haplotype, and the size of the circles is proportional to the frequency of each haplotype. The black squares are the mean vectors and represent putative haplotypes inferred from the analysis. The traces correspond to evolutionary steps. The map showing the geographic distribution of each mtDNA haplogroup was generated using R v4.1.085 and data available in the Harvard Geospatial Library database (https://hgl.harvard.edu/), and edited in Adobe Photoshop CC v. 2017.1.1.

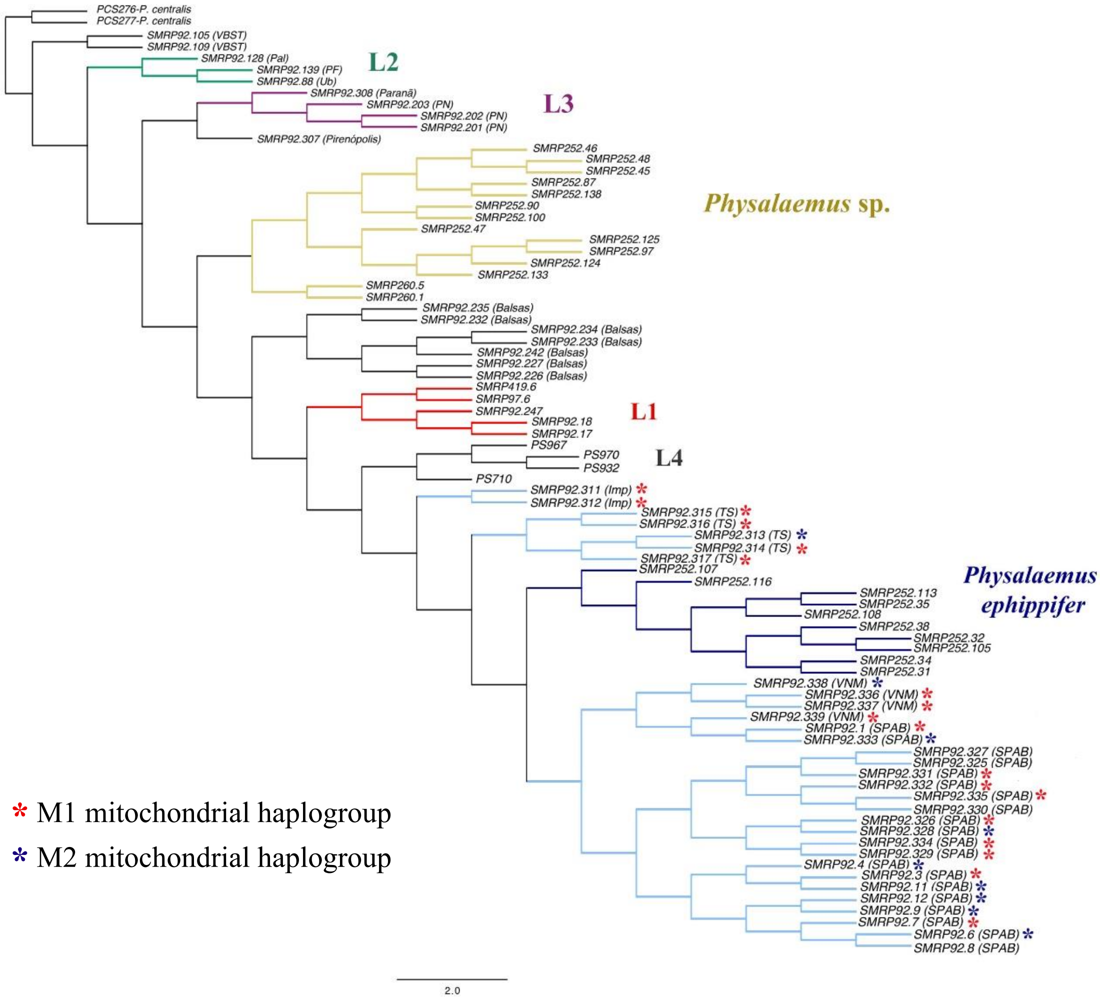

Supplementary Figure S3. Phylogenetic relationships inferred by RAxML analysis of the full 3RAD dataset (83 individuals, 2,757,035 SNPs). Red and blue asterisks indicate specimens from SPAB, VNM, TS, and Imp belonging to the mitochondrial haplogroups M1 and M2, respectively.

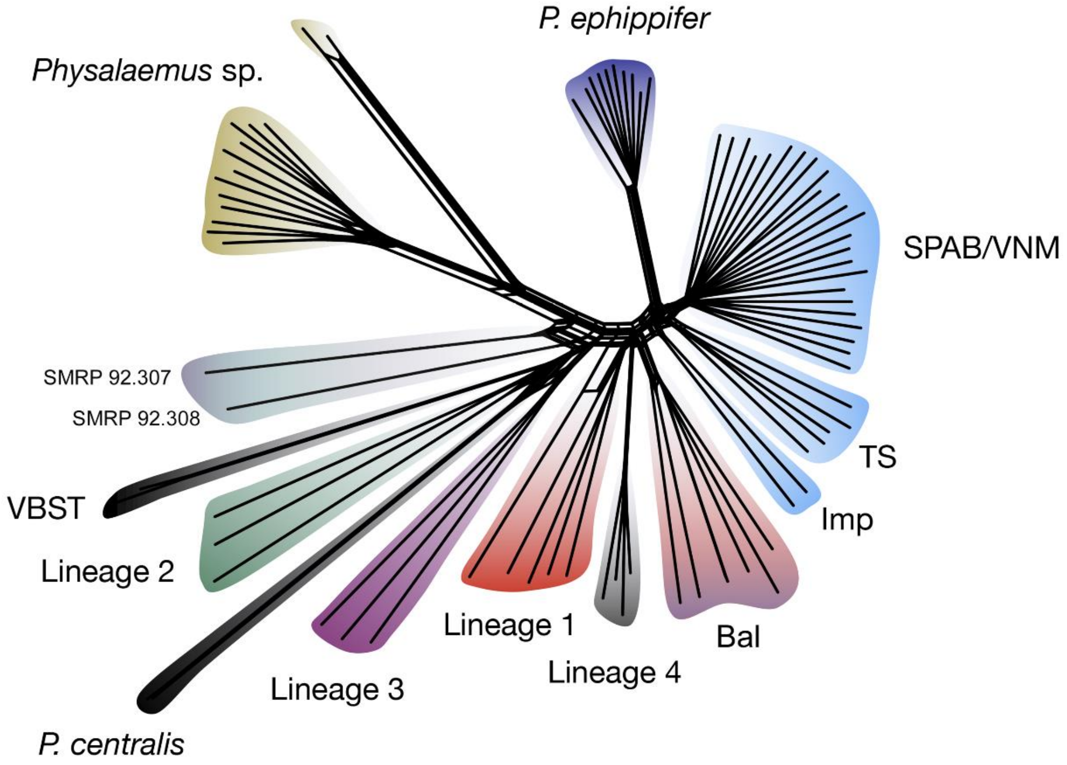

Supplementary Figure S4. NeighborNet phylogenetic network inferred from 3RAD dataset (83 individuals, 205,030 SNPs).

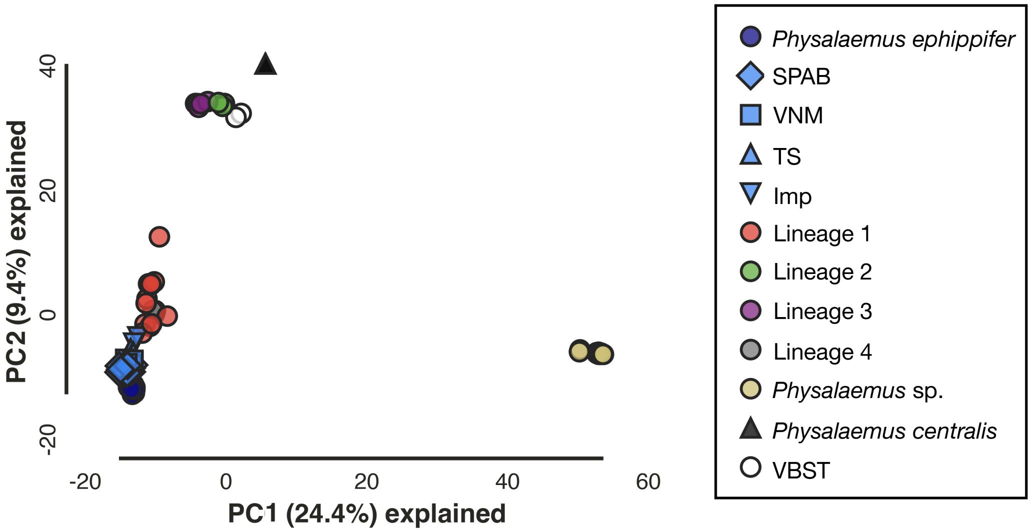

Supplementary Figure S5. Principal component analysis (PCA) of our complete 3RAD dataset (83 individuals, 20,224 SNPs).

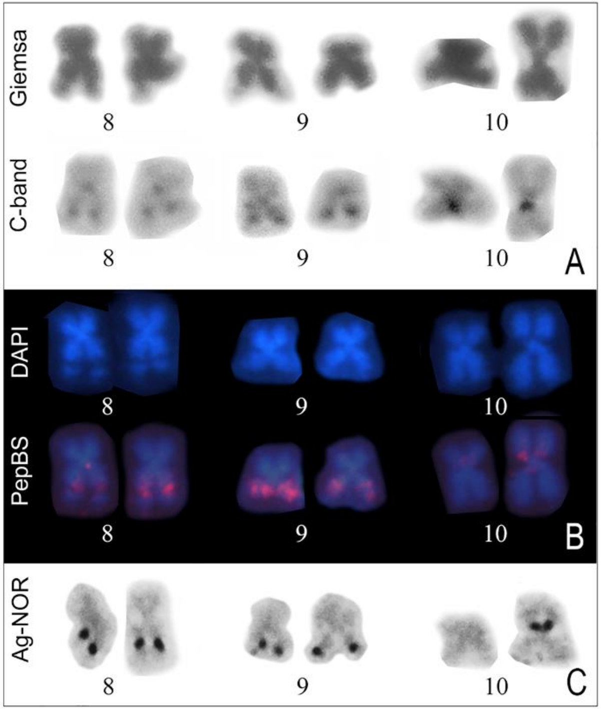

Supplementary Figure S6. NOR-bearing chromosomes 8, 9, and 10 of the specimen SMRP 92.226 from Bal. A. Chromosomes sequentially subjected to Giemsa-staining and C-banding. B. Chromosomes hybridized to a PepBS probe. C. Chromosomes subjected to the Ag-NOR method.
