# Supplementary material for: Multiple contact zones and karyotypic evolution in a neotropical frog species complex
Source: Sci Rep. 2024 Jan 11;14:1119. doi: 10.1038/s41598-024-51421-z (PMC10784582; doi:10.1038/s41598-024-51421-z)
Supplement: Supplementary file 2 — Supplementary Information 1. [file 41598_2024_51421_MOESM2_ESM.pdf]

Cytogenetic analysis of specimens from Porto Nacional (L3)

We expanded the cytogenetic analysis of specimens from Porto Nacional-TO, which belong to L3 lineage, aiming to verify whether NOR-bearing chromosomes 7 and 10 similar to those found in the specimens SMRP 92.232 and 92.226 from Balsas (Bal) are present in L3. Chromosome preparations obtained using the same protocol cited in the main text and were subjected to 10% Giemsa staining and the Ag-NOR method[1]. Fluorescence in situ hybridization (FISH) was used to map nucleolar ribosomal DNA (rDNA) using the rDNA probe HM 123[2] and the protocol described by Viegas-Péquignot[3].

One chromosome 7 with a pericentromeric NOR in the long arm was found in the specimens SMRP 92.76 and SMRP 92.83 (Figure 1 – left image). Chromosome 10 with a pericentromeric NOR in the short arm was present in homozygosis in the specimens SMRP 92.83 and SMRP 92.204 (Figure 1 – right image). These NOR-bearing chromosomes are very similar to those found in specimens from Bal (i.e., SMRP 92.226 and SMRP 92.232; see Figure 10).

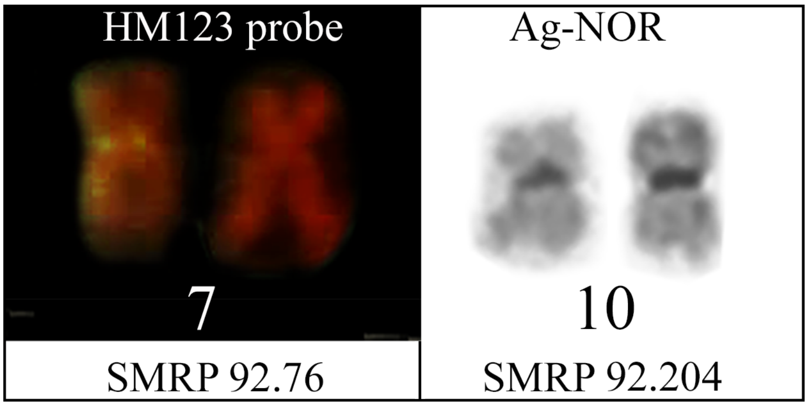

Figure 1. NOR-bearing chromosomes 7 (left) and 10 (right) from specimens of L3 (SMRP 92.76 and SMRP 92.204) hybridized to an rDNA probe (HM123 probe) or silver impregnated by the Ag-NOR method.

References

1. Howell, W.M., Black, D.A. Controlled silver staining of nucleolus organizer regions with a protective colloidal developer: a 1-step method. Experientia **36**, 1014-1015 (1980).
2. Meunier-Rotival, M. *et al.* Isolation and organization of calf ribosomal DNA. Nucleic Acids Res. **6**, 2109-2123 (1979).
3. Viegas-Péquignot, E. In situ hybridization to chromosomes with biotinylated probes. in *In situ Hyridization, A Practical Approach* 137–158 (Wilkinson, D., 1992).
