# Supplementary material for: Multiple contact zones and karyotypic evolution in a neotropical frog species complex
Source: Sci Rep. 2024 Jan 11;14:1119. doi: 10.1038/s41598-024-51421-z (PMC10784582; doi:10.1038/s41598-024-51421-z)
Supplement: Supplementary file 3 — Supplementary Table S1. [file 41598_2024_51421_MOESM3_ESM.pdf]

Supplementary Table S1. Identification, voucher number, and locality of the specimens included in the phylogenetic analyses (based on mitochondrial sequences and RAD markers) and analyzed cytogenetically. The specimens for which we provided new data in this work are highlighted in blue. *Physalaemus atim* Brasileiro and Haddad, 2015 (*[1]) was identified as *Physalaemus* sp. in Lourenço et al.[1]. GenBank accession numbers of all the mitochondrial DNA sequences (12S-tRNA$_{Val}$-16S gene sequences) and the number of RAD loci anlyzed per specimen are provided. CFBH: Collection "Célio F. B. Haddad", Departamento de Zoologia, I.B., Universidade Estadual Paulista (UNESP), Rio Claro-SP, Brazil; CTMZ: Coleção de Tecido do Museu de Zoologia da USP, São Paulo-SP, Brazil; HUFMA: Coleção de Herpetologia da Universidade Federal do Maranhão, São Luís-MA, Brazil; IIBP: Instituto de Investigación Biológica del Paraguay; LGE: Laboratorio de Genética Evolutiva, Instituto de Biología Subtropical (CONICET-UNaM), Posadas, Misiones, Argentina; MNHN: Museo Nacional de Historia Natural, Montevideo, Uruguay; MNRJ: Museu Nacional, Rio de Janeiro-RJ, Brazil; MPEG: Museu Paraense Emílio Goeldi, Belém-PA, Brazil; MZUSP: Museu de Zoologia da USP, São Paulo-SP, Brazil; PS: Collection Pablo Suárez; SMRP: Collection of tissue and chromosome preparation "Shirlei Maria Recco Pimentel", Universidade Estadual de Campinas (UNICAMP), Campinas-SP, Brazil; ZUEC: Museu de Zoologia "Prof. Adão José Cardoso", Universidade Estadual de Campinas (UNICAMP), Campinas-SP, Brazil. *[2]: Lourenço et al. [1]. *[3]: Ron et al. [2]. *[4]: Nascimento et al. [3].

Taxon identity	Voucher	Sample number in tissue collection	Specimens locality	GenBank accession numbers of mtDNA (ref.)	Number of RAD loci analyzed	Specimens analyzed cyto genetically (se
***Physalaemus cuvieri* Clade**						
***Physalaemus cuvieri* Group**						
Physalaemus albifrons	MNRJ 24228	SMRP 74.21	Vassouras, Barreirinhas, State of Maranhão, Brazil	KP146009 (*[2])		
Physalaemus albifrons	CFBH 16137	CFBH-T 5357	Viçosa do Ceará, State of Ceará, Brazil	KP146010 (*[2])		
Physalaemus albonotatus	ZUEC 16219	SMRP 263.15	Lambari D'Oeste, State of Mato Grosso, Brazil	KP146050 (*[2])		
Physalaemus albonotatus		CTMZ 05462	Tangará da Serra, State of Mato Grosso, Brazil	KP146055 (*[2])		
Physalaemus albonotatus		CTMZ 05460	Tangará da Serra, State of Mato Grosso, Brazil	KP146056 (*[2])		
Physalaemus albonotatus	IIBP 1047	IIBP 1047	Estancia Pirá Potrero, Amambay, Paraguay	KP146053 (*[2])		
Physalaemus albonotatus	IIBP 864	IIBP 864	Cerrados del Tagatiyá, Concepción, Paraguay	KP146054 (*[2])		
*Physalaemus atim**[1]	CFBH 12512	CFBH-T 3756	Campo Limpo de Goiás, State of Goiás, Brazil	MH507401/02 (*[2])		
Physalaemus sp. (aff. *albonotatus*)	IIBP 730	IIBP 730	Estancia Ybú, Concepción, Paraguay	KP146057 (*[2])		
Physalaemus sp. (aff. *albonotatus*)	LGE 8871	LGE 8871	Villa Lanús, Misiones, Argentina	KP146060 (*[2])		
Physalaemus sp. (aff. *albonotatus*)	LGE 8872	LGE 8872	Ituzaingó, Corrientes, Argentina	KP146058 (*[2])		
Physalaemus sp. (aff. *albonotatus*)	LGE 8873	LGE 8873	Estancia El Oscuro, Corrientes, Argentina	KP146059 (*[2])		
Physalaemus sp. (aff. *albonotatus*)	DCC-NB 19		4.5 km SE Resistencia, Chaco, Argentina	DQ337210 (*[3])		
Physalaemus centralis	ZUEC 13697	SMRP 96.11	Vitória Brasil, State of São Paulo, Brazil	KP146061 (*[2])		

Physalaemus centralis	ZUEC 13375	SMRP 92.77	Porto Nacional, State of Tocantins, Brazil	KP146062 (*[2])	
Physalaemus centralis	ZUEC 17905	SMRP 419.4	B.Sta.Terezinha, Areia Funda, Alagoinhas, State of Bahia, Brazil	MK241498 (*[4])	
Physalaemus centralis	ZUEC 13101	SMRP 92.26	Urbano Santos, State of Maranhão, Brazil	OR078427	
Physalaemus centralis	PCS276	PS786	Canaã dos Carajás, State of Pará, Brazil (6º23'56" S / 50º22'08" W)	OR078428	15283
Physalaemus centralis	PCS277	PS787	Canaã dos Carajás, State of Pará, Brazil (6º23'56" S / 50º22'08" W)		13468
Physalaemus cuqui	LGE 8874	LGE 8874	2.5 km SE from Aguas Blancas, Salta, Argentina	KP146071 (*[2])	
Physalaemus sp.	ZUEC 18190	SMRP 252.90	Alenquer, State of Pará, Brazil	MK241508 (*[4])	31669
Physalaemus sp.	ZUEC 18185	SMRP 252.87	Monte Alegre, State of Pará, Brazil	MK241509 (*[4])	34190
Physalaemus sp.	ZUEC 22708	SMRP 252.138	Monte Alegre, State of Pará, Brazil		33730
Physalaemus sp.	ZUEC 18188	SMRP 252.88	Óbidos, State of Pará, Brazil	MK241510 (*[4])	
Physalaemus sp.	ZUEC 18196	SMRP 252.97	Óbidos, State of Pará, Brazil		36871
Physalaemus sp.	ZUEC 18203	SMRP 252.100	Óbidos, State of Pará, Brazil	MK241507 (*[4])	33981
Physalaemus sp.	ZUEC 22694	SMRP 252.124	Óbidos, State of Pará, Brazil		32599
Physalaemus sp.	ZUEC 22695	SMRP 252.125	Óbidos, State of Pará, Brazil		34617
Physalaemus sp.	ZUEC 22701	SMRP 252.131	Óbidos, State of Pará, Brazil	MK241503 (*[4])	
Physalaemus sp.	ZUEC 22702	SMRP 252.132	Óbidos, State of Pará, Brazil	MK241504 (*[4])	
Physalaemus sp.	ZUEC 22703	SMRP 252.133	Óbidos, State of Pará, Brazil	MK241505 (*[4])	32573
Physalaemus sp.	ZUEC 22704	SMRP 252.134	Óbidos, State of Pará, Brazil	MK241506 (*[4])	
Physalaemus sp.	ZUEC 17591	SMRP 252.44	Prainha, State of Pará, Brazil	MK241512 (*[4])	
Physalaemus sp.	ZUEC 17592	SMRP 252.45	Prainha, State of Pará, Brazil		31323
Physalaemus sp.	ZUEC 17593	SMRP 252.46	Prainha, State of Pará, Brazil	MK241511 (*[4])	32924
Physalaemus sp.	ZUEC 17594	SMRP 252.47	Prainha, State of Pará, Brazil		36372
Physalaemus sp.	ZUEC 17595	SMRP 252.48	Prainha, State of Pará, Brazil		33990
Physalaemus sp.	ZUEC 17600	SMRP 260.1	Viruá National Park, State of Roraima, Brazil	MK241513 (*[4])	26168
Physalaemus sp.	ZUEC 17604	SMRP 260.5	Viruá National Park, State of Roraima, Brazil	MK241514 (*[4])	29820
"*Physalaemus cuvieri*" (Lineage 1A)	ZUEC 17886	SMRP 92.273	Areia Funda, Alagoinhas, State of Bahia, Brazil	KP146011 (*[2])	
"*Physalaemus cuvieri*" (Lineage 1A)	ZUEC 17897	SMRP 92.284	Caruaru, State of Pernambuco, Brazil	KP146012 (*[2])	
"*Physalaemus cuvieri*" (Lineage 1A)	ZUEC 17912	SMRP 419.11	Macaíba, State of Rio Grande do Norte, Brazil	OR005495	
"*Physalaemus cuvieri*" (Lineage 1B)	ZUEC 13082	SMRP 97.5	Crateús, State of Ceará, Brazil	KP146015 (*[2])	
"*Physalaemus cuvieri*" (Lineage 1B)	ZUEC 13083	SMRP 97.8	Crateús, State of Ceará, Brazil	KP146013 (*[2])	

"*Physalaemus cuvieri*" (Lineage 1B)	ZUEC 13088	SMRP 97.13	Crateús, State of Ceará, Brazil	KP146014 (*[2])	
"*Physalaemus cuvieri*" (Lineage 1B)	ZUEC 13083	SMRP 97.6	Crateús, State of Ceará, Brazil		27031
"*Physalaemus cuvieri*" (Lineage 1B)	ZUEC 13088	SMRP 97.11	Crateús, State of Ceará, Brazil	OR005493	
"*Physalaemus cuvieri*" (Lineage 1B)	ZUEC 13089	SMRP 97.12	Crateús, State of Ceará, Brazil	OR005494	
"*Physalaemus cuvieri*" (Lineage 1B)	ZUEC 13093	SMRP 92.19	Urbano Santos, State of Maranhão, Brazil	OR005490	
"*Physalaemus cuvieri*" (Lineage 1B)	ZUEC 13094	SMRP 92.20	Urbano Santos, State of Maranhão, Brazil	OR005486	
"*Physalaemus cuvieri*" (Lineage 1B)	ZUEC 13102	SMRP 92.27	Urbano Santos, State of Maranhão, Brazil	OR005491	
"*Physalaemus cuvieri*" (Lineage 1B)	ZUEC 13103	SMRP 92.28	Urbano Santos, State of Maranhão, Brazil	OR005492	
"*Physalaemus cuvieri*" (Lineage 1B)	ZUEC 13107	SMRP 92.32	Urbano Santos, State of Maranhão, Brazil	OR005487	
"*Physalaemus cuvieri*" (Lineage 1B)	ZUEC 11939	SMRP 92.33	Urbano Santos, State of Maranhão, Brazil	OR005488	
"*Physalaemus cuvieri*" (Lineage 1B)		SMRP 92.36	Urbano Santos, State of Maranhão, Brazil	OR005489	
"*Physalaemus cuvieri*" (Lineage 1B)	ZUEC 13092	SMRP 92.17	Urbano Santos, State of Maranhão, Brazil	KP146020 (*[2])	26004
"*Physalaemus cuvieri*" (Lineage 1B)	ZUEC 13093	SMRP 92.18	Urbano Santos, State of Maranhão, Brazil	KP146018 (*[2])	27827
"*Physalaemus cuvieri*" (Lineage 1B)	ZUEC 13105	SMRP 92.30	Urbano Santos, State of Maranhão, Brazil	KP146019 (*[2])	
"*Physalaemus cuvieri*" (Lineage 1B)	ZUEC 17899	SMRP 92.286	Araruna, State of Paraíba, Brazil	OR005483	
"*Physalaemus cuvieri*" (Lineage 1B)	ZUEC 17900	SMRP 92.287	Araruna, State of Paraíba, Brazil	OR005484	
"*Physalaemus cuvieri*" (Lineage 1B)	ZUEC 17901	SMRP 92.288	Araruna, State of Paraíba, Brazil	OR005485	
"*Physalaemus cuvieri*" (Lineage 1B)	ZUEC 17907	SMRP 419.6	Araruna, State of Paraíba, Brazil	KP146017 (*[2])	25434
"*Physalaemus cuvieri*" (Lineage 1B)	ZUEC 17908	SMRP 419.7	Macaíba, State of Rio Grande do Norte, Brazil	OR005481	

“*Physalaemus cuvieri*” (Lineage 1B)	ZUEC 17909	SMRP 419.8	Macaíba, State of Rio Grande do Norte, Brazil	OR005482		
“*Physalaemus cuvieri*” (Lineage 1B)	ZUEC 17887	SMRP 92.274	Campina Grande, State of Paraíba, Brazil	OR005480		
“*Physalaemus cuvieri*” (Lineage 1B)	HUFMA 884	SMRP 92.247	São Luís, State of Maranhão, Brazil	KP146021 (*2)	25493	
“*Physalaemus cuvieri*” (L1B x L3)	ZUEC 17516	SMRP 92.226	Balsas, State of Maranhão, Brazil	KP146022 (*2)	30516	male
“*Physalaemus cuvieri*” (L1B x L3)	ZUEC 17517	SMRP 92.227	Balsas, State of Maranhão, Brazil	KP146016 (*2)	28639	
“*Physalaemus cuvieri*” (L1B x L3)	ZUEC 17518	SMRP 92.228	Balsas, State of Maranhão, Brazil	OR005469		
“*Physalaemus cuvieri*” (L1B x L3)		SMRP 92.229	Balsas, State of Maranhão, Brazil	OR005470		male
“*Physalaemus cuvieri*” (L1B x L3)	ZUEC 17520	SMRP 92.230	Balsas, State of Maranhão, Brazil	OR005471		
“*Physalaemus cuvieri*” (L1B x L3)	ZUEC 17522	SMRP 92.232	Balsas, State of Maranhão, Brazil	OR005472	30861	male
“*Physalaemus cuvieri*” (L1B x L3)	ZUEC 17523	SMRP 92.233	Balsas, State of Maranhão, Brazil		35066	
“*Physalaemus cuvieri*” (L1B x L3)	ZUEC 17524	SMRP 92.234	Balsas, State of Maranhão, Brazil	OR005473	31736	
“*Physalaemus cuvieri*” (L1B x L3)	ZUEC 17525	SMRP 92.235	Balsas, State of Maranhão, Brazil	OR005474	35614	
“*Physalaemus cuvieri*” (L1B x L3)	ZUEC 17526	SMRP 92.236	Balsas, State of Maranhão, Brazil	OR005475		
“*Physalaemus cuvieri*” (L1B x L3)	ZUEC 17532	SMRP 92.242	Balsas, State of Maranhão, Brazil	OR005476	33327	
“*Physalaemus cuvieri*” (L1 x *P. ephippifer*)	MNRJ 24255	SMRP 92.1	São Pedro da Água Branca, State of Maranhão, Brazil	OR005460	31364	male
“*Physalaemus cuvieri*” (L1 x *P. ephippifer*)	MNRJ 24257	SMRP 92.3	São Pedro da Água Branca, State of Maranhão, Brazil	OR005461	34425	female
“*Physalaemus cuvieri*” (L1 x *P. ephippifer*)	MNRJ 24258	SMRP 92.4	São Pedro da Água Branca, State of Maranhão, Brazil	OR005463	33426	female
“*Physalaemus cuvieri*” (L1 x *P. ephippifer*)	MNRJ 24259	SMRP 92.5	São Pedro da Água Branca, State of Maranhão, Brazil	OR005464		
“*Physalaemus cuvieri*” (L1 x *P. ephippifer*)	MNRJ 24260	SMRP 92.6	São Pedro da Água Branca, State of Maranhão, Brazil	OR005466	33958	male

"*Physalaemus cuvieri*" (L1 x *P. ephippifer*)	MNRJ 24261	SMRP 92.7	São Pedro da Água Branca, State of Maranhão, Brazil	OR005462	32874	
"*Physalaemus cuvieri*" (L1 x *P. ephippifer*)	MNRJ 24262	SMRP 92.8	São Pedro da Água Branca, State of Maranhão, Brazil		35425	male
"*Physalaemus cuvieri*" (L1 x *P. ephippifer*)	MNRJ 24263	SMRP 92.9	São Pedro da Água Branca, State of Maranhão, Brazil	OR005465	35380	male
"*Physalaemus cuvieri*" (L1 x *P. ephippifer*)	MNRJ 24265	SMRP 92.11	São Pedro da Água Branca, State of Maranhão, Brazil	OR005468	35627	male
"*Physalaemus cuvieri*" (L1 x *P. ephippifer*)	MNRJ 24266	SMRP 92.12	São Pedro da Água Branca, State of Maranhão, Brazil	OR005467	36787	
"*Physalaemus cuvieri*" (L1 x *P. ephippifer*)	HUFMA 2288	SMRP 92.325	São Pedro da Água Branca, State of Maranhão, Brazil	OR005446	29597	male
"*Physalaemus cuvieri*" (L1 x *P. ephippifer*)	HUFMA 2289	SMRP 92.326	São Pedro da Água Branca, State of Maranhão, Brazil	OR005447	29519	male
"*Physalaemus cuvieri*" (L1 x *P. ephippifer*)	HUFMA 2290	SMRP 92.327	São Pedro da Água Branca, State of Maranhão, Brazil	OR005448	24178	female
"*Physalaemus cuvieri*" (L1 x *P. ephippifer*)	HUFMA 2291	SMRP 92.328	São Pedro da Água Branca, State of Maranhão, Brazil	OR005449	32895	male
"*Physalaemus cuvieri*" (L1 x *P. ephippifer*)	HUFMA 2292	SMRP 92.329	São Pedro da Água Branca, State of Maranhão, Brazil	OR005450	30098	male
"*Physalaemus cuvieri*" (L1 x *P. ephippifer*)	HUFMA 2293	SMRP 92.330	São Pedro da Água Branca, State of Maranhão, Brazil		35064	male
"*Physalaemus cuvieri*" (L1 x *P. ephippifer*)	HUFMA 2294	SMRP 92.331	São Pedro da Água Branca, State of Maranhão, Brazil	OR005451	33998	male
"*Physalaemus cuvieri*" (L1 x *P. ephippifer*)	HUFMA 2295	SMRP 92.332	São Pedro da Água Branca, State of Maranhão, Brazil	OR005452	34448	male
"*Physalaemus cuvieri*" (L1 x *P. ephippifer*)	HUFMA 2296	SMRP 92.333	São Pedro da Água Branca, State of Maranhão, Brazil	OR005453	30520	male
"*Physalaemus cuvieri*" (L1 x *P. ephippifer*)	HUFMA 2297	SMRP 92.334	São Pedro da Água Branca, State of Maranhão, Brazil	OR005454	32330	male
"*Physalaemus cuvieri*" (L1 x *P. ephippifer*)	HUFMA 2298	SMRP 92.335	São Pedro da Água Branca, State of Maranhão, Brazil	OR005455	34255	male
"*Physalaemus cuvieri*" (L1 x *P. ephippifer*)	HUFMA 2299	SMRP 92.336	Vila Nova dos Martírios, State of Maranhão, Brazil	OR005456	35019	male
"*Physalaemus cuvieri*" (L1 x *P. ephippifer*)	HUFMA 2300	SMRP 92.337	Vila Nova dos Martírios, State of Maranhão, Brazil	OR005457	34341	male
"*Physalaemus cuvieri*" (L1 x *P. ephippifer*)	HUFMA 2301	SMRP 92.338	Vila Nova dos Martírios, State of Maranhão, Brazil	OR005458	37950	male

"*Physalaemus cuvieri*" (L1 x *P. ephippifer*)	HUFMA 2302	SMRP 92.339	Vila Nova dos Martírios, State of Maranhão, Brazil	OR005459	34499	male
"*Physalaemus cuvieri*" (L1 x *P. ephippifer*)	ZUEC 24592	SMRP 92.313	Trecho Seco - São Francisco do Brejão, State of Maranhão, Brazil	OR005441	25673	
"*Physalaemus cuvieri*" (L1 x *P. ephippifer*)	ZUEC 24591	SMRP 92.314	Trecho Seco - São Francisco do Brejão, State of Maranhão, Brazil	OR005442	29367	
"*Physalaemus cuvieri*" (L1 x *P. ephippifer*)		SMRP 92.315	Trecho Seco - São Francisco do Brejão, State of Maranhão, Brazil	OR005443	28388	
"*Physalaemus cuvieri*" (L1 x *P. ephippifer*)		SMRP 92.316	Trecho Seco - São Francisco do Brejão, State of Maranhão, Brazil	OR005444	28650	
"*Physalaemus cuvieri*" (L1 x *P. ephippifer*)		SMRP 92.317	Trecho Seco - São Francisco do Brejão, State of Maranhão, Brazil	OR005445	30547	
"*Physalaemus cuvieri*" (L1 x *P. ephippifer*)		SMRP 92.311	Imperatriz, State of Maranhão, Brazil	OR005439	31691	
"*Physalaemus cuvieri*" (L1 x *P. ephippifer*)		SMRP 92.312	Imperatriz, State of Maranhão, Brazil	OR005440	28369	
"*Physalaemus cuvieri*" (Lineage 2)		SMRP 92. 318	Rio Verde, State of Goiás, Brazil	OR005438		
"*Physalaemus cuvieri*" (L2 x L3)	ZUEC 20391	SMRP 92.307	Pirenópolis, State of Goiás, Brazil	OR005479	20100	
"*Physalaemus cuvieri*" (Lineage 2)	ZUEC 20381	SMRP 92.297	Serra do Cipó, State of Minas Gerais, Brazil	OR005477		
"*Physalaemus cuvieri*" (Lineage 2)	ZUEC 20383	SMRP 92.299	Três Marias, State of Minas Gerais, Brazil	OR005478		
"*Physalaemus cuvieri*" (Lineage 2)	ZUEC 14631	SMRP 92.180	Chapada dos Guimarães, State of Mato Grosso, Brazil	KP146025 (*[2])		
"*Physalaemus cuvieri*" (Lineage 2)	ZUEC 14623	SMRP 92.172	Chapada dos Guimarães, State of Mato Grosso, Brazil	KP146026 (*[2])		
"*Physalaemus cuvieri*" (Lineage 2)	MZUSP134232	CTMZ 01623	Embu, State of São Paulo, Brazil	KP146029 (*[2])		
"*Physalaemus cuvieri*" (Lineage 2)	ZUEC 14687	SMRP 92.164	Nova Itapirema, State of São Paulo, Brazil	KP146035 (*[2])		
"*Physalaemus cuvieri*" (Lineage 2)	ZUEC 12355	SMRP 92.64	Nova Itapirema, State of São Paulo, Brazil	KP146030 (*[2])		
"*Physalaemus cuvieri*" (Lineage 2)	ZUEC 14681	SMRP 92.158	Nova Itapirema, State of São Paulo, Brazil	KP146032 (*[2])		
"*Physalaemus cuvieri*" (Lineage 2)	ZUEC 14634	SMRP 92.101	Palestina, State of São Paulo, Brazil	KP146028 (*[2])		

"*Physalaemus cuvieri*" (Lineage 2)	ZUEC 14635	SMRP 92.102	Palestina, State of São Paulo, Brazil	KP146034 (*2)	
"*Physalaemus cuvieri*" (Lineage 2)	ZUEC 13670	SMRP 92.127	Palmeiras, State of Bahia, Brazil	KP146045 (*2)	
"*Physalaemus cuvieri*" (Lineage 2)	ZUEC 13671	SMRP 92.128	Palmeiras, State of Bahia, Brazil	KP146044 (*2)	13640
"*Physalaemus cuvieri*" (Lineage 2)	ZUEC 14648	SMRP 92.139	Passo Fundo, State of Rio Grande do Sul, Brazil	KP146040 (*2)	15739
"*Physalaemus cuvieri*" (Lineage 2)	ZUEC 14649	SMRP 92.140	Passo Fundo, State of Rio Grande do Sul, Brazil	KP146038 (*2)	
"*Physalaemus cuvieri*" (Lineage 2)	ZUEC 14657	SMRP 92.216	Passo Fundo, State of Rio Grande do Sul, Brazil	KP146039 (*2)	
"*Physalaemus cuvieri*" (Lineage 2)	LGE 8875	LGE 8875	Puerto Iguazú, Misiones, Argentina	KP146037 (*2)	
"*Physalaemus cuvieri*" (Lineage 2)	CFBH 6442		Rio Claro, State of São Paulo, Brazil	KP146033 (*2)	
"*Physalaemus cuvieri*" (Lineage 2)	ZUEC 13366	SMRP 92.88	Uberlândia, State of Minas Gerais, Brazil	KP146027 (*2)	16574
"*Physalaemus cuvieri*" (Lineage 2)	ZUEC 13368	SMRP 92.90	Uberlândia, State of Minas Gerais, Brazil	KP146041 (*2)	
"*Physalaemus cuvieri*" (Lineage 2)	ZUEC 14714	SMRP 92.184	Vitória da Conquista, State of Bahia, Brazil	KP146046 (*2)	
"*Physalaemus cuvieri*" (Lineage 2)	ZUEC 14715	SMRP 92.185	Vitória da Conquista, State of Bahia, Brazil	KP146042 (*2)	
"*Physalaemus cuvieri*" (Lineage 2)	ZUEC 14729	SMRP 92.199	Vitória da Conquista, State of Bahia, Brazil	KP146043 (*2)	
"*Physalaemus cuvieri*" (Lineage 2)	ZUEC 14667	SMRP 92.97	Vitória Brasil, State of São Paulo, Brazil	KP146031 (*2)	
"*Physalaemus cuvieri*" (Lineage 2)	ZUEC 14669	SMRP 92.99	Vitória Brasil, State of São Paulo, Brazil	KP146036 (*2)	
"*Physalaemus cuvieri*" (Lineage 2)	ZUEC 14670	SMRP 92.100	Vitória Brasil, State of São Paulo, Brazil	KP146024 (*2)	
"*Physalaemus cuvieri*" (Lineage 3)	ZUEC 14691	SMRP 92.200	Porto Nacional, State of Tocantins, Brazil	KP146047 (*2)	
"*Physalaemus cuvieri*" (Lineage 3)	ZUEC 14692	SMRP 92.201	Porto Nacional, State of Tocantins, Brazil		22537
"*Physalaemus cuvieri*" (Lineage 3)	ZUEC 14693	SMRP 92.202	Porto Nacional, State of Tocantins, Brazil	KP146049 (*2)	21888

“*Physalaemus cuvieri*” (Lineage 3)	ZUEC 14694	SMRP 92.203	Porto Nacional, State of Tocantins, Brazil		21989
“*Physalaemus cuvieri*” (Lineage 3)	ZUEC 13374	SMRP 92.76	Porto Nacional, State of Tocantins, Brazil	KP146048 (*2)	
“*Physalaemus cuvieri*” (Lineage 3)	ZUEC 14695	SMRP 92.204	Porto Nacional, State of Tocantins, Brazil	OR005434	
“*Physalaemus cuvieri*” (VBST clade)	ZUEC 17563	SMRP 92.105	Vila Bela Santíssima Trindade, State of Mato Grosso	OR005433	18526
“*Physalaemus cuvieri*” (VBST clade)	ZUEC 17567	SMRP 92.109	Vila Bela Santíssima Trindade, State of Mato Grosso		16998
“*Physalaemus cuvieri*” (Lineage 3)	ZUEC 20390	SMRP 92.306	Paranã, State of Tocantins, Brazil	OR005435	
“*Physalaemus cuvieri*” (Lineage 3)	ZUEC 20392	SMRP 92.308	Paranã, State of Tocantins, Brazil	OR005436	22251
“*Physalaemus cuvieri*” (Lineage 3)	ZUEC 20393	SMRP 92.309	Paranã, State of Tocantins, Brazil	OR005437	
“*Physalaemus cuvieri*” (Lineage 4)	MPEG 40216	PS710	Control I, Marabá, State of Pará, Brazil (5º49'40" S / 50º31'37" W)	OR005429	32571
“*Physalaemus cuvieri*” (Lineage 4)		PS932	Parauapebas, State of Pará, Brazil (6°19'43.23"S 49°56'6.00"W)	OR005430	21327
“*Physalaemus cuvieri*” (Lineage 4)		PS944	Parauapebas, State of Pará, Brazil (6°19'43.23"S 49°56'6.00"W)	OR005431	
“*Physalaemus cuvieri*” (Lineage 4)		PS967	Parauapebas, State of Pará, Brazil (6°19'43.23"S 49°56'6.00"W)	OR005432	20920
“*Physalaemus cuvieri*” (Lineage 4)		PS970	Parauapebas, State of Pará, Brazil (6°19'43.23"S 49°56'6.00"W)		34791
Physalaemus ephippifer	ZUEC 13704	SMRP 252.6	Belém, State of Pará, Brazil	KP146003 (*2)	
Physalaemus ephippifer	ZUEC 13737	SMRP 252.40	Belém, State of Pará, Brazil	KP146004 (*2)	
Physalaemus ephippifer	ZUEC 21357	SMRP 252.107	Santa Bárbara, State of Pará, Brazil		35309
Physalaemus ephippifer	ZUEC 21358	SMRP 252.108	Santa Bárbara, State of Pará, Brazil		39557
Physalaemus ephippifer	ZUEC 21355	SMRP 252.105	Santa Bárbara, State of Pará, Brazil	MK241499 (*4)	32727
Physalaemus ephippifer	ZUEC 21363	SMRP 252.113	Santa Bárbara, State of Pará, Brazil	MK241500 (*4)	36954
Physalaemus ephippifer	ZUEC 21366	SMRP 252.116	Santa Bárbara, State of Pará, Brazil	MK241501 (*4)	43249
Physalaemus ephippifer	ZUEC 13729	SMRP 252.31	Belém, State of Pará, Brazil		43179
Physalaemus ephippifer	ZUEC 13730	SMRP 252.32	Belém, State of Pará, Brazil		45449
Physalaemus ephippifer	ZUEC 13732	SMRP 252.34	Belém, State of Pará, Brazil		40095
Physalaemus ephippifer	ZUEC 13733	SMRP 252.35	Belém, State of Pará, Brazil		43182
Physalaemus ephippifer	ZUEC 13736	SMRP 252.38	Belém, State of Pará, Brazil		39853

Physalaemus ephippifer		PS555	Parque Ambiental Utinga, State of Pará, Brazil	OR005428
Physalaemus erikae	CFBH 32463	CFBH-T 16267	Uruçuca, State of Bahia, Brazil	KP146008 (*[2])
Physalaemus fischeri	MR 005		Calabozo, Guárico, Venezuela	DQ337211 (*[3])
Physalaemus kroyeri	CFBH 23652	CFBH-T 11432	Faz. Santo Onofre e Canabrava, Maracás, State of Bahia, Brazil	KP146005 (*[2])
Physalaemus kroyeri	ZUEC 17481	SMRP 352.2	Ilhéus, State of Bahia, Brazil	KP146006 (*[2])
Physalaemus kroyeri	ZUEC 17904	SMRP 419.3	Alagoinhas, State of Bahia, Brazil	KP145923/33 (*[2])
Physalaemus kroyeri	ZUEC 17911	SMRP 419.10	Bom Conselho, State of Pernambuco, Brazil	KP146007 (*[2])
***Physalaemus biligonigerus* Group**				
Physalaemus marmoratus	ZUEC 13399	SMRP 43.48	São José do Rio Preto, state of São Paulo, Brazil	KP146083 (*[2])
***Physalaemus gracilis* Group**				
Physalaemus barrioi	ZUEC 18146	SMRP 303.1	Serra da Bocaina, state of São Paulo, Brazil	KP146067 (*[2])
***Physalaemus henselii* Group**				
Physalaemus fernandezae	LGE 8876	LGE 8876	Punta Lara, Buenos Aires, Argentina	KP146068 (*[2])
***Physalaemus olfersii* Group**				
Physalaemus feioi	ZUEC 16247	SMRP 247.1	Viçosa, state of Minas Gerais, Brazil	KP146080 (*[2])
***Physalaemus signifer* Clade**				
Physalaemus nattereri	ZUEC 17506	SMRP 58.18	Três Lagoas, State of Mato Grosso do Sul, Brazil	KP146103 (*[2])

References

1. Lourenço, L. B. *et al.* Phylogeny of frogs from the genus *Physalaemus* (Anura, Leptodactylidae) inferred from mitochondrial and nuclear gene sequences. *Mol. Phyl. Evol.* **92**, 204–216 (2015).
2. Ron, S. R. *et al.* Phylogeny of the túngara frog genus *Engystomops* (=*Physalaemus pustulosus* species group; Anura: Leptodactylidae). *Mol. Phylogenet. Evol.* **39**, 392–403 (2006).
3. Nascimento, J. *et al.* Extensive cryptic diversity within the *Physalaemus cuvieri–Physalaemus ephippifer* species complex (Amphibia, Anura) revealed by cytogenetic, mitochondrial, and genomic markers. *Front. Genet.* **10**, 719 (2019).
