# Supplementary material for: Multiple contact zones and karyotypic evolution in a neotropical frog species complex
Source: Sci Rep. 2024 Jan 11;14:1119. doi: 10.1038/s41598-024-51421-z (PMC10784582; doi:10.1038/s41598-024-51421-z)
Supplement: Supplementary file 4 — Supplementary Table S2. [file 41598_2024_51421_MOESM4_ESM.pdf]

Table S2. Summary statistics for each 3RAD data sample produced by ipyrad.

	Number of raw reads	Number of reads after filtering	Heterozygosity (error)	Number of retained loci
PCS_276	10746582	10719093	0.013(0.003)	15283
PCS_277	5560312	5544504	0.011(0.004)	13468
PS_710	10076372	10052191	0.017(0.002)	32571
PS_932	4247079	4239017	0.015(0.003)	21327
PS_967	3825552	3818350	0.015(0.003)	20920
PS_970	10743728	10715967	0.017(0.002)	34791
SMRP_252_100	19717146	19697964	0.017(0.002)	33981
SMRP_252_105	22624868	22605219	0.018(0.002)	32727
SMRP_252_107	27817882	27796842	0.017(0.001)	35309
SMRP_252_108	27232377	27211425	0.017(0.001)	39557
SMRP_252_113	8739715	8722256	0.017(0.002)	36954
SMRP_252_116	11243451	11219559	0.017(0.002)	43249
SMRP_252_124	19459707	19438825	0.018(0.002)	32599
SMRP_252_125	20201239	20183173	0.016(0.002)	34617
SMRP_252_133	21064675	21045067	0.017(0.001)	32573
SMRP_252_138	20004835	19985163	0.016(0.001)	33730
SMRP_252_31	12200189	12176622	0.017(0.002)	43179
SMRP_252_32	14079169	14052156	0.018(0.002)	45449
SMRP_252_34	12995885	12978013	0.018(0.002)	40095
SMRP_252_35	11569040	11541708	0.017(0.002)	43182
SMRP_252_38	12320607	12301012	0.017(0.002)	39853
SMRP_252_45	20386628	20364029	0.018(0.002)	31323
SMRP_252_46	20892944	20868454	0.016(0.002)	32924
SMRP_252_47	24787215	24764059	0.016(0.001)	36372
SMRP_252_48	18412881	18392978	0.018(0.002)	33990
SMRP_252_87	18612749	18595179	0.016(0.002)	34190
SMRP_252_90	17969837	17950486	0.017(0.002)	31669
SMRP_252_97	18555996	18537925	0.016(0.002)	36871
SMRP_260_1	17079202	17059722	0.017(0.002)	26168
SMRP_260_5	24860739	24839443	0.015(0.001)	29820
SMRP_419_6	7644361	7632214	0.016(0.002)	25434
SMRP_92_1	20980553	20961521	0.018(0.002)	31364
SMRP_92_105	12466199	12440856	0.017(0.002)	18526
SMRP_92_109	12565967	12544392	0.017(0.002)	16998
SMRP_92_11	27200834	27178862	0.018(0.001)	35627
SMRP_92_12	22937932	22917657	0.018(0.001)	36787
SMRP_92_128	21751976	21733142	0.020(0.002)	13640
SMRP_92_139	22662229	22641414	0.018(0.002)	15739
SMRP_92_17	21534804	21519185	0.018(0.002)	26004
SMRP_92_18	21713104	21697904	0.017(0.002)	27827
SMRP_92_201	20855694	20834416	0.017(0.002)	22537
SMRP_92_202	19433032	19417103	0.017(0.002)	21888
SMRP_92_203	23025171	23004093	0.018(0.002)	21989
SMRP_92_226	23399112	23380956	0.017(0.001)	30516
SMRP_92_227	19258098	19239895	0.020(0.002)	28639
SMRP_92_232	10097342	10078204	0.017(0.002)	30861
SMRP_92_233	9126737	9105808	0.017(0.002)	35066
SMRP_92_234	9338561	9318616	0.017(0.002)	31736
SMRP_92_235	11221217	11194576	0.018(0.002)	35614
SMRP_92_242	6277754	6263049	0.016(0.002)	33327
SMRP_92_247	20421621	20402950	0.018(0.002)	25493

SMRP_92_3	22375201	22353766	0.018(0.002)	34425
SMRP_92_307	4533898	4520322	0.015(0.002)	20100
SMRP_92_308	5064333	5049220	0.016(0.002)	22251
SMRP_92_311	26745496	26725552	0.018(0.002)	31691
SMRP_92_312	19083301	19070928	0.016(0.002)	28369
SMRP_92_313	9072886	9065685	0.014(0.002)	25673
SMRP_92_314	17729907	17715800	0.016(0.002)	29367
SMRP_92_315	15823851	15810437	0.017(0.002)	28388
SMRP_92_316	16995889	16981989	0.016(0.002)	28650
SMRP_92_317	21941453	21924445	0.018(0.002)	30547
SMRP_92_325	3117971	3111698	0.016(0.002)	29597
SMRP_92_326	3874638	3868417	0.015(0.002)	29519
SMRP_92_327	1814364	1809759	0.017(0.003)	24178
SMRP_92_328	4581679	4571737	0.017(0.002)	32895
SMRP_92_329	3583019	3576019	0.015(0.002)	30098
SMRP_92_330	9521645	9507582	0.017(0.002)	35064
SMRP_92_331	8089005	8076953	0.016(0.002)	33998
SMRP_92_332	7262401	7248446	0.018(0.002)	34448
SMRP_92_333	7710215	7698564	0.017(0.002)	30520
SMRP_92_334	8123293	8111189	0.016(0.002)	32330
SMRP_92_335	6844174	6831532	0.017(0.002)	34255
SMRP_92_336	7881147	7866700	0.017(0.002)	35019
SMRP_92_337	9044457	9029376	0.018(0.002)	34341
SMRP_92_338	10649016	10632129	0.018(0.002)	37950
SMRP_92_339	7825082	7810744	0.017(0.002)	34499
SMRP_92_4	22140440	22116550	0.019(0.002)	33426
SMRP_92_6	8043544	8027366	0.017(0.002)	33958
SMRP_92_7	20270722	20251403	0.019(0.002)	32874
SMRP_92_8	28123034	28101405	0.018(0.001)	35425
SMRP_92_88	17776168	17761634	0.016(0.002)	16574
SMRP_92_9	21753439	21736437	0.017(0.002)	35380
SMRP_97_6	31356068	31329228	0.020(0.001)	27031
